# The two pore potassium channel THIK‐1 regulates NLRP3 inflammasome activation

**DOI:** 10.1002/glia.24174

**Published:** 2022-03-30

**Authors:** Samuel Drinkall, Catherine B. Lawrence, Bernadino Ossola, Samuel Russell, Clare Bender, Nicola B. Brice, Lee A. Dawson, Michael Harte, David Brough

**Affiliations:** ^1^ Division of Pharmacy & Optometry, School of Health Sciences, Faculty of Biology, Medicine and Health University of Manchester, Manchester Academic Health Science Centre Manchester UK; ^2^ Division of Neuroscience and Experimental Psychology, School of Biological Sciences, Faculty of Biology, Medicine and Health University of Manchester, Manchester Academic Health Science Centre Manchester UK; ^3^ Cerevance Ltd Cambridge UK; ^4^ The Lydia Becker Institute of Immunology and Inflammation University of Manchester Manchester UK; ^5^ Geoffrey Jefferson Brain Research Centre, The Manchester Academic Health Science Centre, Northern Care Alliance NHS Group University of Manchester Manchester UK

**Keywords:** inflammasome, inflammation, interleukin‐1, NLRP3, potassium channel, THIK‐1

## Abstract

The NLRP3 (NLR family, pyrin domain containing 3) inflammasome is a multi‐protein complex responsible for the activation of caspase‐1 and the subsequent cleavage and activation of the potent proinflammatory cytokines IL‐1β and IL‐18, and pyroptotic cell death. NLRP3 is implicated as a driver of inflammation in a range of disorders including neurodegenerative diseases, type 2 diabetes, and atherosclerosis. A commonly reported mechanism contributing to NLRP3 inflammasome activation is potassium ion (K^+^) efflux across the plasma membrane. Identification of K^+^ channels involved in NLRP3 activation remains incomplete. Here, we investigated the role of the K^+^ channel THIK‐1 in NLRP3 activation. Both pharmacological inhibitors and cells from THIK‐1 knockout (KO) mice were used to assess THIK‐1 contribution to macrophage NLRP3 activation in vitro. Pharmacological inhibition of THIK‐1 inhibited caspase‐1 activation and IL‐1β release from mouse bone‐marrow‐derived macrophages (BMDMs), mixed glia, and microglia in response to NLRP3 agonists. Similarly, BMDMs and microglia from THIK‐1 KO mice had reduced NLRP3‐dependent IL‐1β release in response to P2X7 receptor activation with ATP. Overall, these data suggest that THIK‐1 is a regulator of NLRP3 inflammasome activation in response to ATP and identify THIK‐1 as a potential therapeutic target for inflammatory disease.

## Main Points

- The NLRP3 inflammasome is implicated as a driver of inflammation in neurodegenerative diseases.
- THIK‐1 regulates ATP‐induced NLRP3 activation in macrophages and microglia.
- THIK‐1 is a potential target for limiting inflammation in disease.

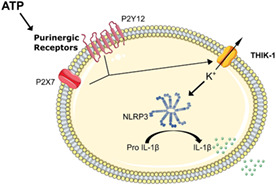

## 1 INTRODUCTION

Inflammation is a response of the immune system to harmful stimuli such as pathogens, damaged cells, and toxic compounds (Medzhitov, 2010), and functions to remove damaging stimuli and initiate healing (Ferrero‐Miliani et al., 2007). Acute inflammatory responses initiate molecular and cellular pathways to minimize injury or infection to restore tissue homeostasis. However, uncontrolled inflammation can become chronic, contributing to a variety of diseases (Lamkanfi & Dixit, 2012). Understanding the mechanisms regulating the inflammatory response is therefore critical to identify new therapeutic targets for limiting damaging inflammation.

Activation of the protease caspase‐1 by inflammasomes is a pro‐inflammatory signaling pathway of the innate immune system (Broz & Dixit, 2016). Upon activation, caspase‐1 drives the processing of pro‐inflammatory cytokine precursors pro‐interleukin (IL)‐1β and pro‐IL‐18 into their mature biologically active forms which subsequently drive inflammatory responses (Dinarello et al., 2012). Of the inflammasomes identified, the inflammasome formed by the sensor NLRP3 (NLR family, pyrin domain containing 3) is the most researched due to its association with a number of inherited and acquired inflammatory diseases (Hoffman et al., 2001; Wen et al., 2012). NLRP3‐dependent inflammation is suggested to drive neuroinflammation in neurodegenerative conditions including Alzheimer's and Parkinson's disease in addition to peripheral diseases such as atherosclerosis, type‐2 diabetes, and others (Wang et al., 2020). Accordingly, there is a great interest in understanding the mechanisms regulating NLRP3 to identify potential therapeutic targets for limiting damaging inflammation.

NLRP3‐inflammasome activation is induced by pathogen or damage associated molecular patterns (PAMPs and DAMPs respectively) (Broz & Dixit, 2016). Due to the structural diversity of NLRP3 activators, it is thought unlikely they directly bind to, and activate, NLRP3. Activators of NLRP3 are reported to indirectly induce NLRP3 activation via altering cellular homeostasis and inducing organelle dysfunction which is in turn sensed by NLRP3 (Seoane et al., 2020). Following activation, NLRP3 nucleates the oligomerization of the adaptor protein apoptosis‐associated speck‐like protein containing a caspase recruitment domain (ASC) (Lu et al., 2014). ASC itself then undergoes oligomerization into inflammasome specks which leads to caspase‐1 recruitment and activation. Caspase‐1 cleaves pro‐IL‐1β and pro‐IL‐18 to active released forms (Dick et al., 2016; Lu et al., 2014). In addition to activation of pro‐inflammatory cytokines, active caspase‐1 also triggers a pro‐inflammatory form of programmed cell death termed pyroptosis via the cleavage of gasdermin D (GSDMD) (Shi et al., 2015). Following cleavage, GSDMD forms pores in the cell membrane which act as a conduit for IL‐1β release (Heilig et al., 2018), and which also leads to cell swelling and nerve injury‐induced protein 1 (NINJ1) dependent cell membrane rupture resulting in the release of pro‐inflammatory intracellular contents (Ding et al., 2016; Kayagaki et al., 2021). Canonical activation of NLRP3 in vitro requires a two‐step activation process. The first “priming” step can be induced by stimulation of Toll‐like receptors which drives the expression of pro‐IL‐1β and NLRP3 (Hornung & Latz, 2010). The second step is NLRP3 inflammasome activation, which can be induced by a range of structurally unrelated stimuli such as the K^+^ ionophore nigericin, extracellular ATP, and crystalline/particulate matter such as silica (Hornung et al., 2008; Mariathasan et al., 2006). Several studies have proposed mechanisms to explain how such a diverse range of stimuli converge on NLRP3 activation. One proposed mechanism is a decrease in intracellular K^+^ (Pétrilli et al., 2007) which is suggested to be important for multiple NLRP3 activating stimuli (Muñoz‐Planillo et al., 2013). However, the mechanism by which K^+^ efflux regulates NLRP3 activation remains unclear. Recent studies have also shown small molecules such as imiquimod and the imidazoquinoline derivative CL097 trigger NLRP3 activation independently of K^+^ efflux (Groß et al., 2016). K^+^ efflux is therefore an important but not universal driver of canonical NLRP3 activation. Studies also suggest an involvement of Cl^−^ channels in NLRP3 activation (Kelley et al., 2019). We have previously shown Cl^−^ efflux can regulate NLRP3‐dependent ASC oligomerization (Green et al., 2018). An alternative K^+^ independent mechanism of NLRP3 activation is also described in human monocytes (Gaidt et al., 2016).

K^+^ channels regulate an array of cellular and immune responses including immune cell proliferation, cell volume regulation, cytokine production and surveillance (Bittner et al., 2010; Bobak et al., 2011; Madry et al., 2018; Meuth et al., 2008). Members of the two‐pore domain K^+^ (K2P) channel family in particular, have recently been implicated with NLRP3 inflammasome activation (Di et al., 2018; Madry et al., 2018). The Two‐pore domain Weak Inwardly rectifying K^+^ channel 2 (TWIK2) has been suggested to facilitate ATP‐induced K^+^ efflux and subsequent NLRP3 activation in macrophages (Di et al., 2018). Furthermore, a recent study identified the K^+^ channel Tandem Pore Domain Halothane‐Inhibited Potassium Channel 1 (THIK‐1) regulates IL‐1β release from hippocampal slices in response to ATP (Madry et al., 2018), suggesting that it may be important for NLRP3 activation. Using pharmacological and genetic approaches we identified the K^+^ channel THIK‐1 as a specific regulator of NLRP3 inflammasome activation in response to extracellular ATP, but not to other NLRP3 activating stimuli. These results suggest THIK‐1 may represent a potential therapeutic target in limiting damaging NLRP3 inflammasome activation in inflammatory disease where ATP signaling is a component.

## 2 MATERIALS AND METHODS

### 2.1 Materials

Pharmacological reagents were obtained from the following manufacturers: Sigma (ML133, quinine, tetraethylammonium chloride (TEA), tetrapentylammonium (TPA), MCC950, lipopolysaccharide (LPS) from *Escherichia coli* O26:B6, and ATP), AdooQ (TRAM‐34 and PAP‐1), Alomone Labs (guangxitoxin‐1E), Tocris (dofetilide), Merck Millipore (Ac‐YVAD‐CMK), U.S Silica (Silica), Life Technologies (DNA [pEF/v5‐His A plasmid empty vector], Lipofectamine 3000), and Invivogen (ultrapure flagellin from *Salmonella typhimurium* and imiquimod). Specific antibodies were used targeting: mouse IL‐1β (AF‐401, R&D), caspase‐1 p10 (EPR16883, Abcam), gasdermin D (ab209845, Abcam), NLRP3 (G‐20B‐0014‐C100, Adipogen), and β‐actin (Sigma). All other materials/reagents were obtained from Sigma unless otherwise stated.

### 2.2 Generation of THIK‐1 knockout mice

The THIK‐1 gene (*kcnk13)* was disrupted by MRC Harwell by using CRISPR/Cas9 to insert a single nucleotide into the wild‐type DNA sequence (Bradley et al., 2012; Brown & Moore, 2012; Pettitt et al., 2009). Insertion resulted in a frameshift mutation in the codon of for amino acid 14. This resulted in a premature stop codon after amino acid 68. The mice were maintained as homozygotes on a C57BL/6 background. To confirm the genotype of the mice Taqman MGB Allelic Discrimination genotyping assays were designed using Primer Express 3.0.1 (Applied Biosystems). Taqman MGB probes were purchased from ThermoFisher & primers were purchased from Sigma Aldrich.

**Table:** 

Primer/probe name	Sequence (5′–3′)
MmKCNK13 SNP genotyping FP	GGTCGGCAGAGCACATCCT
MmKCNK13 SNP genotyping RP	CTGCAACTCCTGCGCTAGCT
MmKCNK13 WT SNP genotyping probe	FAM‐CACCTGAACGAGGAC‐MGB
MmKCNK13 KO SNP genotyping probe	VIC‐CACCTGAATCGAGGAC‐MGB

Lysates were prepared from ear snips using Extract‐N‐Amp Tissue PCR Kit from Sigma (XNAT2R). qPCR was run in 384 plates with the following thermocycler conditions: 60°C × 30 s, 95°C × 10 min, then 40 cycles at 95°C × 15 s, 60°C × 1 min, lastly one cycle at 60°C × 30 s.

### 2.3 Primary and immortalized murine BMDM preparation

Primary bone marrow‐derived macrophage (pBMDM) cells were prepared from adult wildtype (WT, C57BL/6) and THIK‐1 knockout (KO) male and female mice aged 6–12 weeks. In brief, bone marrow was flushed from femurs, red blood cells were lysed before culturing the remaining cells in Dulbecco's Modified Eagle's Medium (DMEM) (Gibco) containing 10% (vol/vol) fetal bovine serum (FBS, Thermo), 100 U ml^−1^ penicillin and 100 μg ml^−1^ streptomycin (PenStrep, Thermo), supplemented with L929 conditioned media (30% vol/vol) or M‐CSF (20 ng ml^−1^). pBMDMs were differentiated for 6–7 days, with extra L929 conditioned or M‐CSF media added on day 3. pBMDMs were then re‐seeded overnight at a density of 1 × 10^6^ cells ml^−1^ prior to experiments. pBMDMs were primed with 1 μg ml^−1^ LPS for 4 h then treated with drug or vehicle (1% [vol/vol] DMSO) in serum free DMEM for 15 min. Following drug incubation, the NLRP3 inflammasome was activated by stimulation with ATP (5 mM) or nigericin (10 μM) for 1 h, imiquimod (75 μM) for 2 h or silica (300 μg ml^−1^). Alternatively, the NLRC4 inflammasome and AIM2 inflammasome were activated by transfection with ultrapure flagellin from *Salmonella typhimurium* (1 μg ml^−1^) or DNA (pEF/v5‐His A plasmid empty vector) (1 μg ml^−1^) respectively for 4 h. Transfections were performed using Lipofectamine 3000 according to the manufacturer's instructions. For priming experiments pBMDMs were treated with drug or vehicle (1% [vol/vol] DMSO) for 15 min prior to priming with 1 μg ml^−1^ LPS for 4 h. Supernatants were removed and analyzed for IL‐1β, IL‐6, and tumor necrosis factor (TNF) content by ELISA (Duoset, R&D Systems). Supernatants and lysates were collected to analyze IL‐1β, caspase‐1, NLRP3, and GSDMD processing by western blot.

Immortalized BMDM (iBMDM) cells were a kind gift from Prof. Clare Bryant (Department of Veterinary Medicine, University of Cambridge). iBMDMs were cultured in DMEM supplemented with 10% (vol/vol) FBS and 1% (vol/vol) PenStrep. Before experiments iBMDMs were seeded overnight at a density of 0.75 × 10^6^ cells ml^−1^ at 37°C. For priming experiments iBMDMs were treated with drug or vehicle (1% [vol/vol] DMSO) for 15 min prior to priming with LPS (1 μg ml^−1^, 4 h). Supernatants were removed and analyzed for IL‐6 and TNF content by ELISA (Duoset, R&D Systems) according to manufacturer's instructions. For inflammasome activation experiments iBMDMs were seeded overnight at a density of 0.75 × 10^6^ cells ml^−1^ at 37°C. Cells were primed with LPS (1 μg ml^−1^, 4 h) then treated with drug or vehicle in serum free DMEM for 15 min or where appropriate media was changed to fresh media containing the indicated isotonic salt solution: control (145 mM NaCl/5 mM KCl), high K^+^ and normal Cl^−^ (150 mM KCl), or high K^+^ and Cl^−^ free solution (150 mM KGluconate) for 15 min. NLRP3 inflammasome activation was then stimulated by adding ATP (5 mM) for 1 h. Supernatants were removed and analyzed for IL‐1β, IL‐6 and TNF content by ELISA. Supernatants and lysates were collected to analyze IL‐1β and NLRP3 protein expression by western blot.

### 2.4 Primary human monocyte and THP‐1 preparation

Fresh blood was isolated from healthy volunteers following approval from Ethics Committee 05/Q0401/108 and 2017–2551‐3945 (University of Manchester). Peripheral blood mononuclear cells (PBMC) were isolated from blood using a 30% Ficoll gradient (Thermo) and centrifugation at 400*g* for 40 min at room temperature. The PBMC layer was separated and washed three times with MACs buffer to remove platelets. Monocytes were positively selected by incubating with CD14+ magnetic microbeads (Miltenyi) for 15 min at 4°C and then passed though LS columns. Cells were pelleted and counted before seeding at 1 × 10^6^ cells ml^−1^ in 96 well plates for immediate use. Human WT and NLRP3 KO THP‐1 cells were cultured in RPMI‐1640 medium supplemented with 10% (vol/vol) FBS, 1% (vol/vol) PenStrep, and 2 mM l‐glutamine and seeded in 96 well plates for immediate use. THP‐1 cells were primed with LPS (1 μg ml^−1^, 4 h) then treated with drug or vehicle (1% DMSO) in serum free media for 15 min. Following drug incubation canonical NLRP3 activation was stimulated with silica (300 μg ml^−1^, 4 h). For alternative NLRP3 activation primary human monocytes and THP‐1 cells were treated with drug or vehicle (DMSO) in serum free media for 15 min before stimulation with LPS for 16 h. Supernatants were removed and analyzed for IL‐1β content by ELISA.

### 2.5 Primary murine mixed glial culture preparation

Murine mixed glial cells were prepared from the brains of C57BL/6 male and female 2‐4‐day old mice that were sacrificed by cervical dislocation under S1. All experimental procedures were performed under Home Office UK project license in accordance with the Animals (Scientific Procedures) Act UK 1986 and approved by the University of Manchester AWERB (Animal Welfare and Ethical Review Body). The brains were isolated, followed by dissection of hemispheres and removal of meninges as previously described (Hoyle et al., 2020). Tissue was homogenized by trituration in DMEM supplemented with 10% FBS and 1% PenStrep. Homogenate was centrifuged at 500*g* for 10 min and the pellet was resuspended in fresh DMEM with 10% (vol/vol) FBS and 1% (vol/vol) PenStrep. Cells were washed at day 5 and media replaced. Media was replaced every 2 days. Cells were seeded at 2 × 10^5^ cells ml^−1^ in 96 well plates and incubated for 2 days before use. Mixed glia were primed with 1 μg ml^−1^ LPS for 4 h then treated with drug or vehicle (1% DMSO [vol/vol]) in serum free DMEM for 15 min Following drug incubation NLRP3 was activated by stimulation with ATP (5 mM, 1 h), nigericin (10 μM, 1 h), imiquimod (75 μM, 2 h) or silica (300 μg ml^−1^, 4 h). Supernatants were collected and analyzed for IL‐1β content by ELISA.

### 2.6 Primary murine adult microglia preparation

WT and THIK‐1 KO C57BL/6 male and female mice aged 6–10 weeks were sacrificed by cervical dislocation under S1. All experimental procedures were performed under Home Office UK project license in accordance with the Animals (Scientific Procedures) Act UK 1986 and approved by the University of Manchester and University of Cambridge AWERB (Animal Welfare and Ethical Review Body). All subsequent steps, unless otherwise stated, were performed at 4°C. Brains were isolated followed by removal of cerebellum and meninges. Brains were minced with a disposable scalpel into 1–3 mm^3^ chunks and digested using a Neural Tissue Dissociation Kit (Miltenyi) according to manufacturer's instructions. The resulting suspension was homogenized using a Dounce tissue grinder with 20 passes of a loose clearance pestle. Myelin was removed from the subsequent single‐cell suspension by centrifuging in 33% Percoll (GE Healthcare) for 10 min at 1000*g* with low break and aspiration of myelin layer. Cells were pelleted by diluting 1:4 in Hanks balanced salt solution without calcium or magnesium (Gibco) and centrifuged for 10 min at 500*g*. Cells were incubated with 10 μl anti‐CD11b magnetic microbeads (Miltenyi) per brain in MACs buffer PBS without calcium or magnesium with 2 mM ethylenediaminetetraacetic acid and 0.5% (wt/vol) bovine serum albumin (BSA) for 15 min at 4°C under slow rotation followed by passing through LS columns (Miltenyi) according to manufacturer's instructions. The remaining microglia suspension was pelleted, counted and spot plated onto 96‐well Cell+ plates (Sarstedt) at a density of 20,000–30,000 cells per well. The plates were left at room temperature for 10 min to allow cell attachment followed by addition of 100 μl culture medium (Dulbecco's modified Eagle's medium/F12 containing 10% (vol/vol) FBS, 1% (vol/vol) PenStrep, and 2 mm glutamine supplemented with IL‐34 (20 ng ml^−1^; R&D Systems) and transforming growth factor‐*β*_1_ (50 ng ml^−1^; Miltenyi). Cells were used at day 8. Microglia were primed with 1 μg ml^−1^ LPS for 4 h then treated with drug or vehicle (1% (vol/vol) DMSO) in serum free media for 15 min Following drug incubation NLRP3 was stimulated with ATP (5 mM, 1 h), nigericin (10 μM, 1 h), imiquimod (75 μM, 2 h) or silica (300 μg ml^−1^, 4 h). For priming experiments microglia were treated with drug or vehicle (1% DMSO) for 15 min prior to priming with LPS (1 μg ml^−1^, 4 h). Supernatants were removed and analyzed for IL‐1β, IL‐6, and TNF content by ELISA.

### 2.7 Cytokine assessment

IL‐1β, IL‐6, and TNF released into culture supernatants were measured using the ELISA DuoSet™ kits (R&D Systems) following manufacturer's instructions.

### 2.8 Western blotting

IL‐1β, caspase‐1, and GSDMD processing in addition to NLRP3 and IL‐1β protein expression were determined by western blotting. Both cell supernatant and cell lysates were collected together and precipitated in deoxycholate containing 20% trichloroacetic acid (Fisher) and washed with acetone followed by air drying at room temperature before concentration in 2x Laemmlii buffer. All samples were separated using Tris‐glycine SDS/PAGE and then transferred using a semi‐dry Transblot Turbo System (Bio‐Rad) at 25 V onto nitrocellulose or PVDF membranes. Membranes were blocked in 5% (wt/vol) BSA (Sigma) in PBS, 1% (vol/vol) Tween 20 (PBST). Following blocking, membranes were incubated with primary antibodies: IL‐1β (1:800), caspase‐1 (1:1000), GSDMD (1:1000), NLRP3 (1:1000), or β‐actin‐peroxidase (1:20000) in 1% (vol/vol) BSA PBST at 4°C overnight. Membranes were then incubated and labeled with HRP‐tagged secondary antibodies (1:1000) in 1% (vol/vol) PBST and visualized with Amersham ECL detection reagent (GE Healthcare). Images of western blots were captured digitally using a G‐Box Chemi XX6 (Syngene).

### 2.9 Lactate dehydrogenase assay

Cell death was quantified in pBMDMs, iBMDMs and mixed glia following treatment by measuring the release of the enzyme lactate dehydrogenase (LDH). This was achieved using the Cytotox‐96 assay (Promega) according to the manufacturer's instructions.

### 2.10 ASC speck imaging

Real‐time ASC speck assays were performed using iBMDMs stably expressing ASC‐mCherry (ASC‐mCherry iBMDMs) (Daniels et al., 2016). ASC‐mCherry iBMDMs were seeded out overnight into 96 well plates at a density of 0.75 × 10^6^ cells ml^−1^ followed by priming for 3 h with 1 μg ml^−1^ LPS. To prevent pyroptosis and loss of ASC specks iBMDMs were pre‐treated with the pan‐caspase inhibitor Ac‐YVAD‐CMK (50 μM) for 30 min prior to microscopy. After priming, cells were pre‐treated with vehicle control (1% [vol/vol] DMSO), TPA (50 μM) or MCC950 (10 μM), or appropriate cells were reperfused with high K^+^ and normal Cl^−^ , or high K^+^ and Cl^−^ free solution for 15 min. Following pre‐treatment, cells were stimulated with ATP (5 mM) or reperfused with Cl^−^ free, K^+^ free, or K^+^ and Cl^−^ free solution where indicated. Images were captured using a 20x/0.61 S Plan Fluor objective at 15‐min intervals and quantified using an Incucyte Zoom System (Essen Bioscience). Comparison of ASC speck formation was analyzed after stimulation for 105, 150, or 165 mins.

### 2.11 YO‐PRO‐1 P2X7 assay

P2X7 receptor‐dependent membrane permeability was determined using the YO‐PRO‐1 fluorescent dye (Rat et al., 2017). iBMDMs were seeded out overnight into 96 well plates at a density of 0.75 × 10^6^ cells ml^−1^ followed by priming for 4 h with 1 μg ml^−1^ LPS. After priming, cells were pretreated with vehicle control (1% [vol/vol] DMSO), TPA (50 μM) or the P2X7 inhibitor oxidized ATP (oATP, 5 mM) before stimulation with ATP (5 mM) for 30 min. Supernatant was removed and cells washed with PBS without calcium or magnesium. 200 μl of YO‐PRO‐1 (2 μM) staining solution (Thermofisher Scientific) was then added to cells and fluorescence measured every 5 min for 30 min.

### 2.12 Statistical analysis

Data are presented as the mean ± standard error of the mean (SEM). Levels of significance accepted were *p < .05, **p < .01, ***p < .001, ****p < .0001. Statistical significance was calculated using GraphPad Prism version 9.2.0. Data with multiple groups were analyzed using a one‐way ANOVA followed by Dunnet's post hoc comparison. Experiments with two independent variables were analyzed using two‐way ANOVA followed by Bonferroni's post hoc correct analysis.

## 3 RESULTS

### 3.1 Potassium channels shared by cultured BMDM and microglia

In order to identify potential K^+^ channels involved in regulating NLRP3 activation in macrophages and microglia we analyzed K^+^ channel RNA expression in both cultured microglia and iBMDM cells from existing datasets (Hoyle et al., 2018). Through quantitative expression analysis of existing RNA expression databases (https://braininflammationgroup-universityofmanchester.shinyapps.io/GrapheneOxide/,https://braininflammationgroup-universityofmanchester.shinyapps.io/NLRP3KOmicroglia) we identified eight different K^+^ channels to be expressed by both microglia and iBMDMs (Supplementary Figure S1). Channels identified included the calcium‐activated K^+^ channel KCa3.1, the inwardly rectifying K^+^ channel Kir2.1, K2P channels TWIK‐2 and THIK‐1 and voltage gated K^+^ channels Kv1.3, 2.1, 4.1, and 11.1.

### 3.2 Pharmacological blockage of two‐pore domain potassium channels inhibited NLRP3 inflammasome activation

To identify K^+^ channels involved in activation of the NLRP3 inflammasome, LPS‐primed (1 μg ml^−1^, 4 h) pBMDMs were incubated for 15 min with K^+^ channel inhibitors TEA (non‐selective K^+^ inhibitor), TRAM‐34 (KCa3.1 inhibitor), TPA (THIK‐1 and TWIK‐2 inhibitor), ML133 (Kir2.1 inhibitor), quinine hydrochloride dihydrate (THIK‐1 and TWIK‐2 inhibitor), Guangxitoxin‐1E (Kv2.1 inhibitor), PAP‐1 (Kv1.3 inhibitor), and dofetilide (Kv11.1 inhibitor). The inhibitors selected inhibited one or more of the K^+^ channels identified above (Supplementary Figure S1), in addition to the non‐specific broad K^+^ channel inhibitor TEA. The NLRP3 inhibitor MCC950 (Coll et al., 2015) was included as a positive control. Inhibitors and concentrations used were based on previously published work (Madry et al., 2018; Nguyen et al., 2017; Paul et al., 2001; Roy et al., 2010; Schaarschmidt et al., 2009; Schmalhofer et al., 2009; Schmitz, 2005). BMDMs were then stimulated with ATP (5 mM, 1 h) to induce NLRP3 activation via the P2X7 receptor (Mariathasan et al., 2006), or with silica (300 μg ml^−1^, 4 h) to stimulate NLRP3 activation via lysosome damage (Hornung et al., 2008). Both ATP‐ and silica‐induced activation of the NLRP3 inflammasome require K^+^ efflux (Muñoz‐Planillo et al., 2013).

**FIGURE 1:**
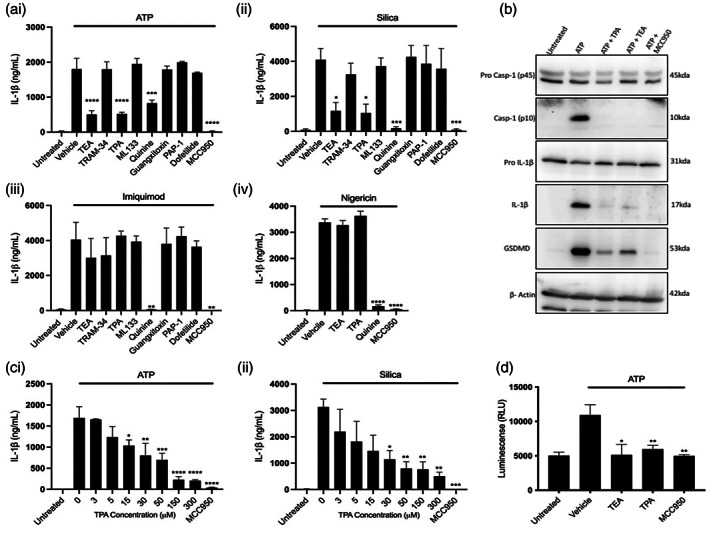
Pharmacological inhibition of two pore domain potassium channels blocks NLRP3 inflammasome activation and IL‐1β processing. (ai–iii) IL‐1β ELISA of the supernatant of pBMDMs primed with LPS (1 μg ml^−1^, 4 h) followed by pretreatment with MCC950 (10 μM) or K^+^ channel inhibitors TEA (50 mM), TRAM‐34 (10 μM), TPA (50 μM), ML‐133 (20 μM), quinine (100 μM), Guangitoxin‐1E (25 nM), PAP‐1 (2 μM), or Dofetilide (1 μM) for 15 min before stimulation with ATP (5 mM, 1 h) (*n* = 4), silica (300 μg ml^−1^, 4 h) (*n* = 5) or imiquimod (75 μM, 2 h) (*n* = 3). (Aiv) IL‐1β ELISA of the supernatant of pBMDMs primed with LPS (1 μg ml^−1^, 4 h) followed by pretreatment with MCC950 (10 μM) or TEA (50 mM), TPA (50 μM), quinine (100 μM), for 15 min before stimulation with nigericin (10 μM, 1 h) (*n* = 4). (b) Caspase‐1, IL‐ β and gasdermin D western blot of total cell lysates (cell lysate + supernatant) from LPS‐primed (1 μg ml^−1^, 4 h) pBMDMs pretreated with vehicle control, TEA (50 mM), TPA (50 μM) or MCC950 (10 μM) for 15 min then stimulated with ATP (5 mM, 1 h). (c) IL‐1β ELISA of the supernatant of pBMDMs primed with LPS (1 μg ml^−1^, 4 h) followed by pretreatment with MCC950 (10 μM) or TPA (3–300 μM) before stimulation with ATP (5 mM, 1 h) (*n* = 5) or silica (300 μg ml^−1^, 4 h) (*n* = 3). (d) Caspase‐1 Glo assay to measure caspase‐1 activity of LPS‐primed (1 μg ml^−1^, 4 h) pBMDMs pretreated with vehicle control, TEA (50 mM), TPA (50 μM) or MCC950 (10 μM) for 15 min then stimulated with ATP (5 mM, 1 h). ****p < .0001, ***p < .001, **p < .01, *p < .05 determined by one‐way ANOVA with Dunnett's post hoc analysis. Values shown are the mean ± SEM

The role of K^+^ channel function in NLRP3 activation was demonstrated by use of the non‐specific K^+^ channel blocker TEA (50 mM) which significantly inhibited IL‐1β release in response to both ATP (Figure 1ai) and silica (Figure 1aii). Blocking K2P channels (including THIK‐1 and TWIK‐2) with TPA (50 μM), or quinine (100 μM) (Lotshaw, 2007; Piechotta et al., 2011), also significantly inhibited ATP‐induced IL‐1β release (Figure 1ai) and silica‐induced IL‐1β release (Figure 1aii). In contrast, the inhibitors blocking KCa3.1 (TRAM‐34, 10 μM), Kir2.1 (ML133, 20 μM), Kv2.1 (Guangxitoxin‐1E, 25 nM), Kv1.3 (PAP‐1, 2 μM) and Kv11.1 (dofetilide, 1 μM) had no impact on IL‐1β release in response to ATP or silica (Figure 1ai,ii, respectively). The Kir2.1 inhibitor ML133 did however significantly reduce cell death in response to ATP, but not silica, which is in contrast to all of the other K^+^ channel inhibitors used, which failed to reduce cell death in response to ATP or silica (Supplementary Figure S2A).

Having established which K^+^ channel inhibitors attenuate IL‐1β release in response to ATP and silica, we next confirmed that the effects of TEA, TPA and quinine were due to their inhibitory action on K^+^ channels, and not an alternative “off target” mechanism. In order to test this we examined the impact of the K^+^ channel inhibitors on NLRP3‐dependent IL‐1β release using the K^+^ efflux‐independent NLRP3 activator imiquimod (Groß et al., 2016). Stimulation of LPS‐primed pBMDMs for 1 h with 75 μM imiquimod induced IL‐1β release, which was abolished by pre‐treatment with quinine (Figure 1aiii). In contrast, TEA, TPA and other inhibitors had no effect on imiquimod‐induced IL‐1β release. Imiquimod‐induced cell death was not significantly affected by treatment with any inhibitor tested (Supplementary Figure S2A). In addition to imiquimod, we investigated the effect of TEA, TPA and quinine on nigericin‐induced NLRP3 activation. Nigericin is a K^+^ ionophore which activates NLRP3 by facilitating K^+^ efflux independently of K^+^ channels (Mariathasan et al., 2006). Stimulation of LPS‐primed pBMDMs for 1 h with 10 μM nigericin induced IL‐1β release which was unaffected by pre‐treatment with TEA or TPA (Figure 1aiv). Pretreatment with quinine also reduced nigericin‐induced IL‐1β release (Figure 1aiv). Nigericin‐induced cell death was not significantly affected by treatment with TPA, TEA or quinine (Supplementary Figure S2A). The effect of ATP, silica, imiquimod and nigericin on IL‐1β release were all NLRP3‐dependent as in all cases IL‐1β release was inhibited by MCC950 (Figure 1a). Taken together these data suggest that the K^+^ channels targeted by TEA and TPA may play a role in ATP‐ and silica‐induced activation of NLRP3. The specific effect of TPA on K^+^ channel dependent NLRP3‐mediated IL‐1β release indicated a role of K2P channels in this pathway. Although only weakly, TEA also inhibits K2P channels (Lotshaw, 2007). Therefore, TEA may also inhibit NLRP3 activation via inhibition of K2P channels. In contrast, since quinine could inhibit NLRP3 activation in response to imiquimod, a K^+^ ‐efflux independent activator, and nigericin, a K^+^ ionophore, suggests quinine may be inhibiting NLRP3 through alternative mechanisms in addition to blocking K^+^ channels.

Western blot analysis showed that ATP‐induced caspase‐1, IL‐1β, and GSDMD processing were all inhibited by TPA and TEA (Figure 1b) further indicating that TPA and TEA are inhibitors of NLRP3 inflammasome activation. TPA inhibited both ATP‐ and silica‐induced IL‐1β release in a concentration dependent manner (Figure 1ci,cii). Caspase‐1 Glo, a quantitative measure of caspase‐1 activity showed that TPA and TEA inhibited caspase‐1 activity in pBMDMs in response to ATP treatment (Figure 1d) further suggesting TPA and TEA as inhibitors of NLRP3 activation. Interestingly, inhibition of NLRP3 activation, caspase‐1 cleavage and subsequent GSDMD cleavage with TPA and TEA in response to ATP failed to inhibit cell death. These results suggest the cell death observed in these studies is not pyroptosis. One potential explanation for these findings is the cell death is not pyroptosis but necrosis as has been described for NLRP3 activating stimuli previously (Cullen et al., 2015).

In addition to NLRP3, other well characterized inflammasomes AIM2 and NLRC4 also drive caspase‐1 cleavage and subsequent IL‐1β release in response to cytosolic DNA and intracellular bacteria respectively (Fernandes‐Alnemri et al., 2009; Schroder & Tschopp, 2010). Previous studies have shown high extracellular K^+^ fails to block AIM2 and NLRC4 inflammasomes (Muñoz‐Planillo et al., 2013; Pétrilli et al., 2007) demonstrating dependence on K^+^ efflux may be a unique feature of the NLRP3 inflammasome. The AIM2 and NLRC4 inflammasomes can be activated by transfection of poly(dA:dT) and flagellin respectively (Baldwin et al., 2017). To determine whether the effect of K2P channel inhibition on IL‐1β release is selective to NLRP3 inflammasome activation, the impact of K^+^ channel inhibition on AIM2 and NLRC4‐dependent IL‐1β release was tested. LPS‐primed pBMDMs were pretreated with K^+^ channel inhibitors as described above. AIM2 or NLRC4 inflammasome activation was then stimulated to induce IL‐1β release by transfecting BMDMs with poly (dA:dT) (1 μg ml^−1^, 4 h) or *salmonella typhimurium* flagellin (1 μg ml^−1^, 4 h). Pretreatment with K^+^ channel inhibitors failed to reduce IL‐1β release or cell death in response to AIM2 or NLRC4 inflammasome activation (Supplementary Figure S3). These data suggest K^+^ channels and specifically K2P channels selectively regulate NLRP3 inflammasome activation without impacting other inflammasomes.

Following the findings that K2P channel inhibition attenuated canonical NLRP3 activation in mouse macrophages we next aimed to determine whether K2P channel inhibition also regulated canonical NLRP3 activation in human immune cells. As observed in mouse BMDMs, stimulation of LPS‐primed THP‐1 monocytes with silica (300 μg mL^−1^, 4 h) induced IL‐1β release which was inhibited by both TEA and TPA (Supplementary Figure S4). IL‐1β release was inhibited in both MCC950 treated THP‐1 cells and NLRP3 KO THP‐1 cells, demonstrating the response was NLRP3 dependent. These data indicate that K2P channels may regulate canonical NLRP3 activation in both murine and human immune cells. In addition to two step canonical NLRP3 activation, LPS alone can stimulate caspase‐1 activation and IL‐1β release in human monocytes (Perregaux et al., 1996). This mechanism has been defined as alternative NLRP3 inflammasome activation and does not require K^+^ efflux (Gaidt et al., 2016). The impact of the K2P channel inhibition on alternative NLRP3 activation in human monocyte THP‐1 and also primary human CD14+ monocytes freshly isolated from healthy donors was therefore tested. Treatment with LPS (1 μg ml^−1^, 16 h) induced alternative NLRP3 activation and subsequent IL‐1β release in both THP‐1 and primary human monocytes which was inhibited by both TEA and TPA (Supplementary Figure S4). Together these data show K^+^ channel inhibitors TEA and TPA inhibit both canonical and alternative NLRP3 activation in human monocytes. The findings that both TEA and TPA inhibit K^+^ efflux independent alternative NLRP3 activation suggests both TEA and TPA can inhibit alternative NLRP3 activation through additional mechanisms independent from preventing K^+^ efflux via K2P channels.

**FIGURE 2:**
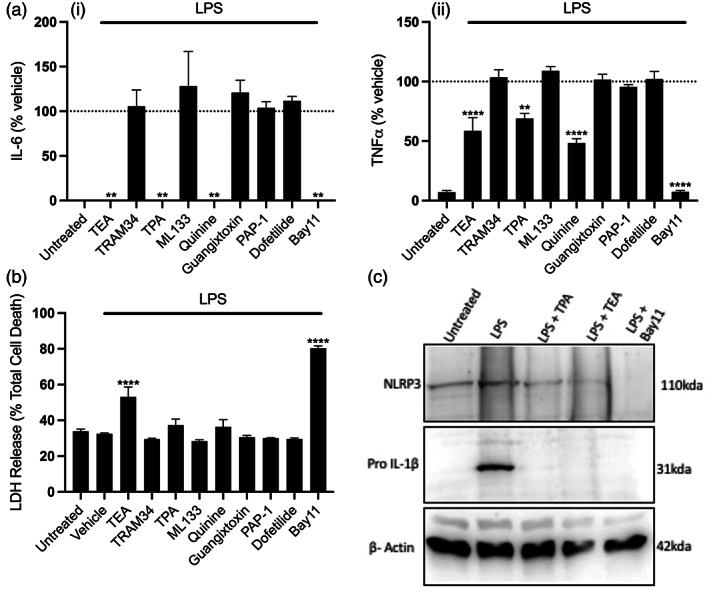
Pharmacological inhibition of two pore potassium channels blocks priming of the NLRP3 inflammasome. (a) IL‐6 (i) and TNF (ii) ELISA and (b) LDH release assay of the supernatant of iBMDMs pretreated with Bay11(10 μM) or K^+^ channel inhibitors TEA (50 mM), TRAM‐34 (10 μM), TPA (50 μM), ML‐133 (20 μM), quinine (100 μM), Guangitoxin‐1E (25 nM), PAP‐1 (2 μM) or Dofetilide (1 μM) for 15 min before priming with LPS (1 μg ml^−1^, 4 h) (*n* = 4). (c) NLRP3 and IL‐1β western blot of the supernatant and total cell lysates respectively of iBMDMs pretreated with TEA (50 mM), TPA (50 μM) or Bay11 (10 μM) for 15 min before priming with LPS (1 μg ml^−1^, 4 h). ****p < .0001, **p < .01 determined by one‐way ANOVA with Dunnett's post hoc analysis. Values shown are the mean ± SEM

We also investigated the impact of K^+^ channel inhibition on the priming step of canonical NLRP3 inflammasome activation. LPS stimulation results in the activation of the transcription factor NF‐κB, which in addition to upregulating NLRP3 and pro‐IL‐1β expression, also upregulates other pro inflammatory cytokines such as IL‐6 and TNF (Bauernfeind et al., 2009; Liu et al., 2017). iBMDMs were pretreated with K^+^ channel inhibitors or the NF‐κB inhibitor Bay11 for 15 min prior to priming with LPS for 4 h. TEA and TPA significantly inhibited both IL‐6 and TNF release in response to LPS (Figure 2ai,ii), and while TEA showed some toxicity this was not the case for TPA (Figure 2b). By western blot we confirmed TPA and TEA inhibited LPS‐induced protein expression of NLRP3 and pro‐IL‐1β (Figure 2c). Together these results show that TPA and TEA inhibit both NLRP3 priming and activation steps, suggesting K2P channels may also play a role in NLRP3 priming as well as activation.

### 3.3 Blocking two‐pore domain potassium channels enhances ASC speck formation but does not trigger caspase‐1 activation

**FIGURE 3:**
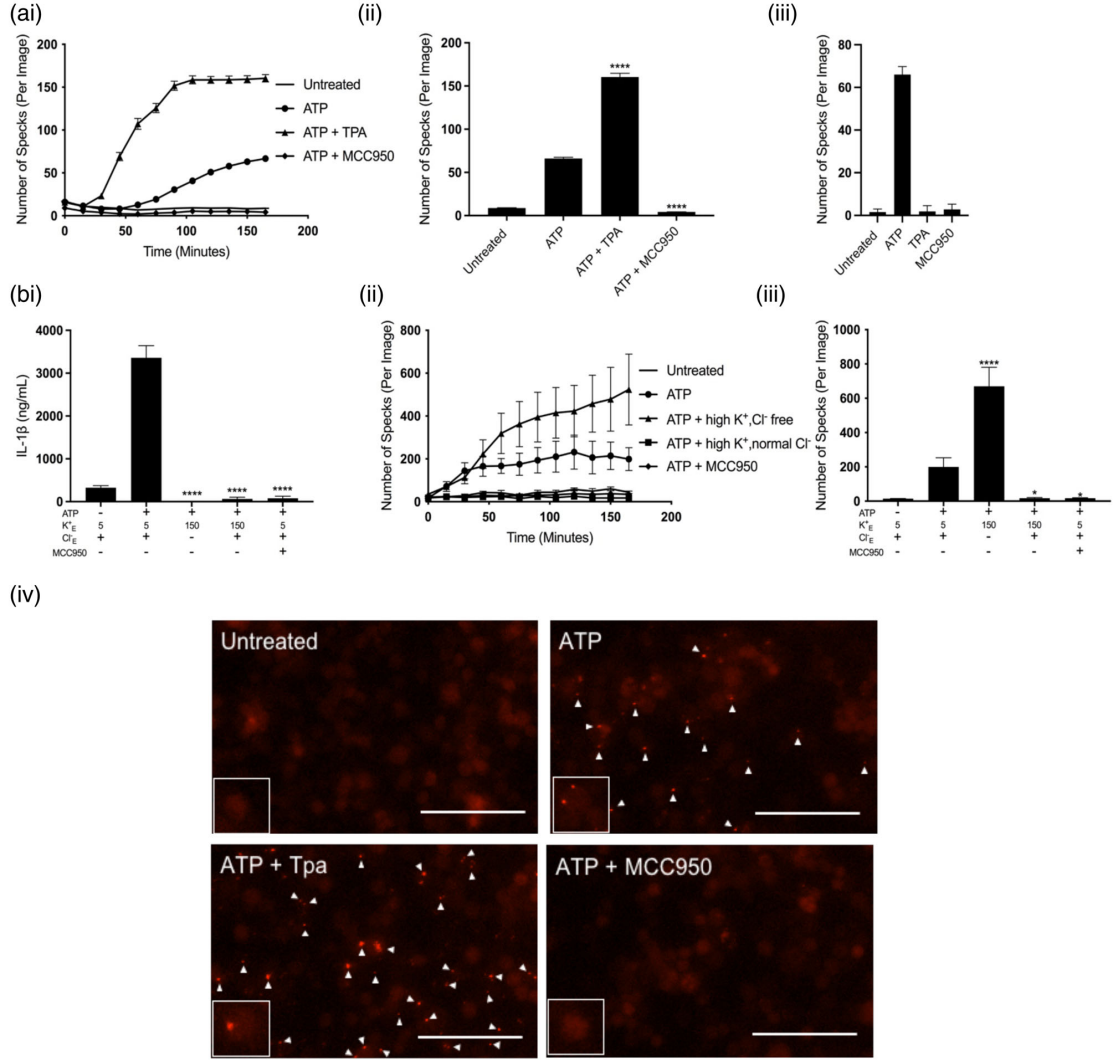
Potassium efflux is required for NLRP3 inflammasome activation but not ASC speck formation in response to ATP. (a, i) ASC speck formation measured in real time and (a, ii) ASC speck formation after 165 min of ATP stimulation from ASC‐mCherry iBMDMs primed with LPS (1 μg ml^−1^, 4 h) followed by pretreatment vehicle control, TPA (50 μM) or MCC950 (10 μM) for 15 min before stimulation with ATP (5 mM) (*n* = 6). (a, iii) ASC speck formation after 165 min from ASC‐mCherry iBMDMS primed with LPS (1 μg ml^−1^, 4 h) followed by treatment with vehicle control, TPA (50 μM) or MCC950 (10 μM) in the absence of ATP (*n* = 6). (b, i) IL‐1β ELISA of the supernatant of iBMDMs primed with LPS (1 μg ml^−1^, 4 h) followed by incubation in a control (145 mM NaCl/ 5 mM KCl), high K^+^ and normal Cl^−^ (150 mM KCl), high K^+^ and Cl^−^ free (150 mM KGluconate) or control and MCC950 (10 μM) solution for 15 min before stimulation with ATP (5 mM, 1 h) (*n* = 6). (b, ii) ASC speck formation measured in real time and (b, iii) ASC speck formation after 165 min of ATP stimulation from iBMDMs stably expressing ASC‐mCherry (ASC‐mCherry iBMDMs) primed with LPS (1 μg ml^−1^, 4 h) followed by incubation in a control (145 mM NaCl/ 5 mM KCl), high K^+^ and normal Cl^−^ (150 mM KCl), high K^+^ and Cl^−^ free (150 mM KGluconate) or control and MCC950 (10 μM) solution for 15 min before stimulation with ATP (5 mM) (*n* = 4). (b, iv) representative images of ASC‐mCherry iBMDMs after 165 min ATP stimulation (scale bar, 50 μm, arrows denote ASC specks). ASC speck experiments were performed in the presence of ac‐YVAD‐CMK (50 μM) to prevent pyroptosis and loss of ASC specks. ****p < .0001, *p < .05 determined by one‐way ANOVA with Dunnett's post hoc analysis. Values shown are the mean ± SEM

Using iBMDMs stably expressing ASC‐mCherry (Daniels et al., 2016), we studied the effect of K^+^ channel inhibitors on ASC speck formation over time. Incubation of LPS‐primed ASC‐mCherry iBMDMs with 5 mM ATP induced the formation of ASC specks which were inhibited by MCC950 (Figure 3ai,ii). TPA evoked a large and significant increase in the number of ASC specks formed in response to ATP. These data reveal ASC speck formation occurs in the presence of concentrations of TPA that inhibited the activation of caspase‐1. We next tested whether TPA alone in the absence of a NLRP3 activating stimulus could induce ASC speck formation. Treatment with TPA alone in LPS‐primed ASC‐mCherry iBMDMs failed to induce ASC speck formation (Figure 3aiii). These data show a dissociation between ASC speck formation which resembles the oligomerization of inflammasome components from activation of the NLRP3 inflammasome into a caspase‐1 activating complex. ASC oligomerization occurred in the presence of the TPA suggesting K2P channel activation may be required to shift inactive preformed ASC specks to active caspase‐1 cleaving complexes.

We previously reported Cl^−^ flux to be required for ASC oligomerization while caspase‐1 activation is K^+^ efflux‐dependent (Green et al., 2018). Having observed that inhibition of K2P channels with TPA increased ASC speck formation in response to ATP in this study, we next wanted to determine whether TPA was enhancing speck formation via blocking K^+^ efflux through K2P channels. We therefore investigated whether blocking K^+^ efflux also enhanced ASC speck formation in response to ATP. To understand the effect of blocking K^+^ efflux on inflammasome activation we performed ion substitution experiments. LPS‐primed iBMDMs were incubated in solutions with normal K^+^ and normal Cl^−^, high K^+^ and Cl^−^ free, or high K^+^ and normal Cl^−^ . High K^+^ will block K^+^ efflux as previously reported (Green et al., 2018). NLRP3 activation was then stimulated with ATP. Incubation with both high K^+^ or high K^+^ and Cl^−^ free completely abolished IL‐1β release in response to ATP (Figure 3bi). These data show that blocking K^+^ efflux is sufficient to block activation of the NLRP3 inflammasome supporting our findings that K^+^ channel inhibitors block NLRP3‐inflammasome activation (Figure 1). We next aimed to investigate the impact of incubating iBMDMs in the above‐mentioned isotonic salt solutions on ASC speck formation. To test the impact of these isotonic salt solutions on ASC speck formation, ASC‐mCherry iBMDMs were primed with LPS and incubated with (a) normal K^+^, normal Cl^−^, (b) high K^+^, normal Cl^−^, or (c) high K^+^, Cl^−^ free solutions. NLRP3 activation was then stimulated with ATP and ASC formation analyzed in real time. Blocking both K^+^ and Cl^−^ efflux with high K^+^ and high Cl^−^ solution completely inhibited the formation of ASC‐specks in response to ATP (Figure 3bii,iii). In contrast, allowing Cl^−^ efflux but blocking K^+^ efflux with high K^+^ and Cl^−^ free solution enhanced ASC speck formation in response to ATP (Figure 3bii). These findings are consistent with our previous report that Cl^−^ efflux serves as an ASC oligomerizing signal while K^+^ efflux is required for NLRP3 activation (Green et al., 2018). These results suggested that blocking K^+^ efflux directly or inhibiting K2P channels inhibited NLRP3‐dependent caspase‐1 activation but enhanced ASC speck formation.

### 3.4 Two pore domain potassium channel inhibition blocks NLRP3 activation in mixed glia and adult microglia

**FIGURE 4:**
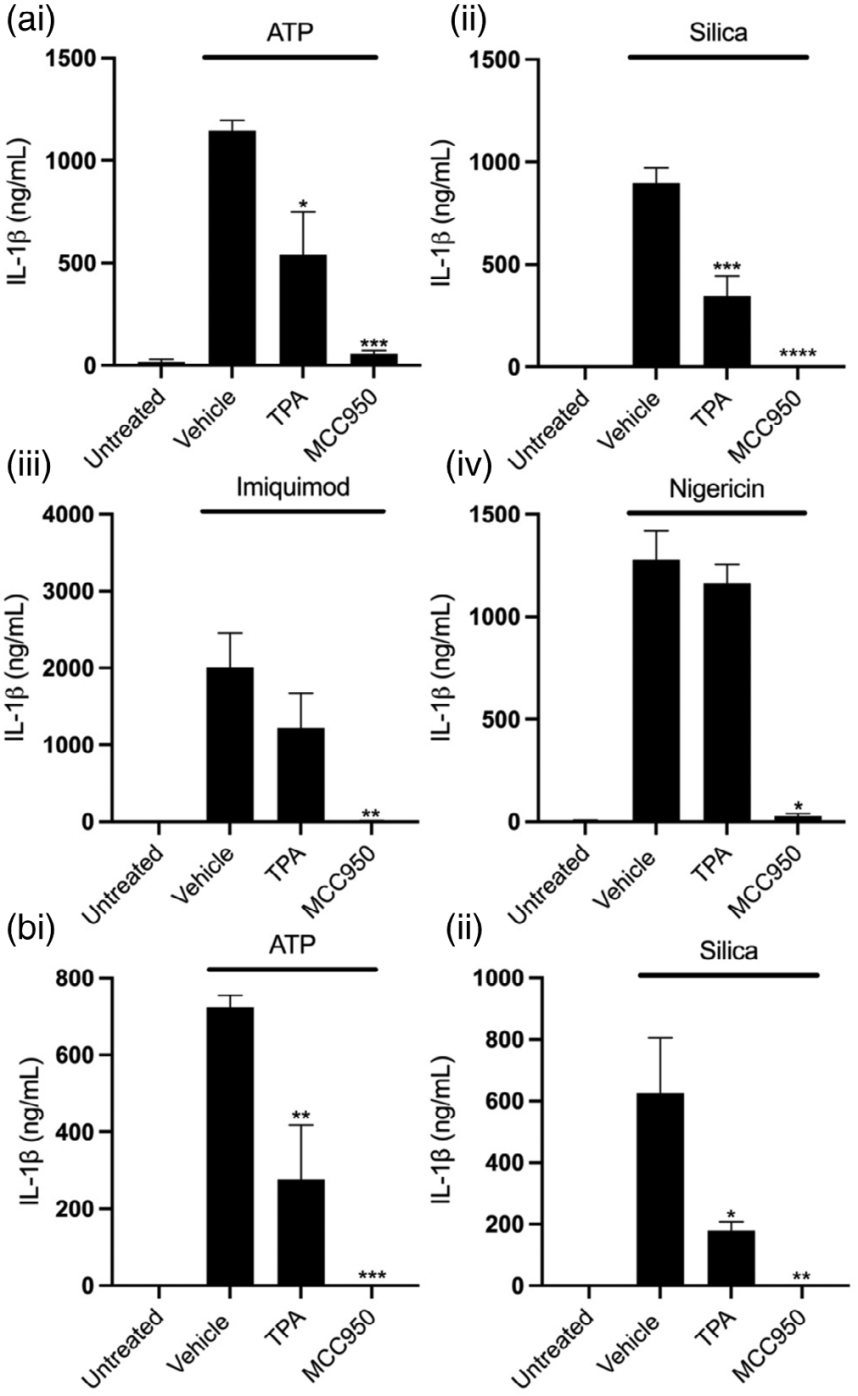
Inhibition of THIK‐1 blocks NLRP3 activation in mixed glia and isolated microglia. (a) IL‐1β ELISA of the supernatant of primary mouse mixed glia primed with LPS (1 μg ml^−1^, 4 h) followed by pretreatment with vehicle control, TPA (50 μM) or MCC950 (10 μM) for 15 min before stimulation with ATP (5 mM, 1 h) (*n* = 5), silica (300 μg ml^−1^,4 h) (*n* = 3), imiquimod (75 μM, 2 h) (*n* = 4) or nigericin (10 μM, 1 h) (*n* = 3). (b) IL‐1β ELISA of the supernatant of mouse primary microglia primed with LPS (1 μg ml^−1^, 4 h) followed by pretreatment with vehicle control, TPA (50 μM) or MCC950 (10 μM) for 15 min before stimulation with ATP (5 mM, 1 h) or silica (300 μg ml^−1^,4 h) (*n* = 3). ****p < .0001, ***p < .001, **p < .01, *p < .05 determined by one‐way ANOVA with Dunnett's post hoc analysis. Values shown are the mean ± SEM

Having observed that TPA inhibited NLRP3 activation in iBMDMs we next sought to determine whether TPA mediated K2P inhibition could block NLRP3 in microglia, a brain resident macrophage cell population. Initially, we investigated the impact of TPA on ATP‐, silica‐, nigericin‐, or imiquimod‐induced NLRP3 activation in primary mouse mixed glial cultures. Supporting our previous findings in BMDMs, TPA also inhibited NLRP3 activation in mixed glial cultures containing both microglia and astrocytes in response to ATP and silica (Figure 4ai,ii). TPA failed to inhibit nigericin or imiquimod induced NLRP3 activation consistent with our findings in BMDMs (Figure 4aiii,iv). TPA had no impact on cell death in response to any NLRP3 stimuli (Supplementary Figure S6). These results show TPA inhibited NLRP3 activation in mixed glia potentially through its ability to block K2P channels. To confirm TPA was inhibiting NLRP3 activation in microglial cells directly we evaluated the effect of TPA on ATP and silica induced NLRP3 activation in isolated adult microglia. We found TPA inhibited IL‐1β release from isolated microglia in response to both ATP and silica (Figure 4b). These results suggest TPA sensitive channels are important for regulating NLRP3 activation within CNS resident microglia as well as peripheral macrophages.

### 3.5 THIK‐1 specifically regulates ATP‐induced NLRP3 inflammasome activation in macrophages

**FIGURE 5:**
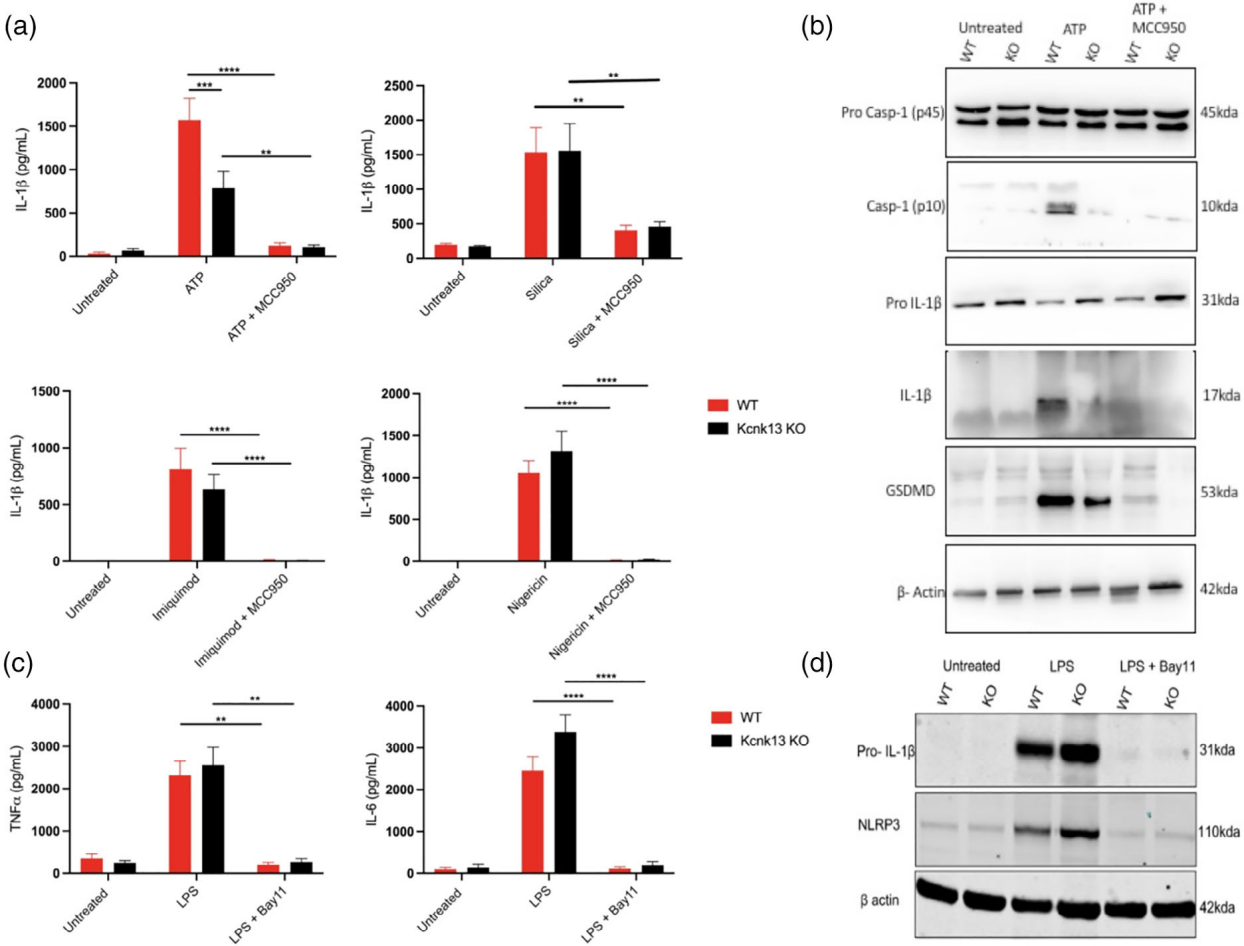
THIK‐1 specifically regulates ATP‐induced NLRP3 activation in bone‐marrow‐derived macrophages. (a) IL‐1β ELISA of the supernatant of primary wild‐type (WT) and Kcnk13 knockout (KO) BMDMs primed with LPS (1 μg ml^−1^, 4 h) followed by pretreatment with MCC950 (10 μM) for 15 min before stimulation with ATP (5 mM, 1 h) (*n* = 7), silica (300 μg ml^−1^,4 h) (*n* = 7), imiquimod (75 μM, 2 h) (*n* = 4) or nigericin (10 μM, 1 h) (*n* = 8). (b) Caspase‐1, IL‐1β and gasdermin D western blot of total cell lysates (cell lysate + supernatant) from LPS‐primed WT and Kcnk13 KO pBMDMs pretreated with vehicle control or MCC950 (10 μM) for 15 min before stimulated with ATP (5 mM, 1 h). (c and d) TNF and IL‐6 ELISA and NLRP3 and IL‐1β western blot of the supernatant and total cell lysates respectively of primary wild‐type (WT) and Kcnk13 knockout (KO) BMDMs pretreated with Bay11(10 μM) for 15 min before priming with LPS (1 μg ml^−1^, 4 h) (*n* = 7). ****p < .0001, ***p < .001, **p < .01 determined by two‐way ANOVA with Bonferroni's post hoc analysis. Values shown are the mean ± SEM

Although our data using TPA suggested K2P channels played a role in NLRP3 activation, K^+^ channel modulators are known to inhibit cellular signaling pathways independently of K^+^ channels (Akopova, 2017; Humphries & Dart, 2015). We therefore utilized genetic approaches to further determine which specific K^+^ channel regulates NLRP3 activation. Of the K2P channel family, cultured BMDMs and microglia had high expression of THIK‐1 and TWIK‐2 channels (Supplementary. Figure S1). Previous research has already shown TWIK‐2 to facilitate ATP‐induced K^+^ efflux and NLRP3 activation (Di et al., 2018). We therefore investigated the impact of THIK‐1 KO on NLRP3 inflammasome activation. To investigate the role of THIK‐1 in macrophage NLRP3 activation, we harvested pBMDMs from WT and THIK‐1 KO mice. THIK‐1 (KCNK13) KO was confirmed by genotyping. WT and THIK‐1 KO BMDMs were primed with LPS and NLRP3 was activated by stimulation with ATP, silica, imiquimod and nigericin. Removal of THIK‐1 specifically inhibited IL‐1β release in response to ATP, and had no effect on the response to silica, imiquimod, or nigericin stimulation (Figure 5a). THIK‐1 KO had no effect on cell death in response to any of the stimuli tested (Supplementary Figure S7). ATP triggers NLRP3 inflammasome activation via activation of the P2X7 receptor (Solle et al., 2001). We therefore wanted to determine whether THIK‐1 regulation of ATP‐induced activation was occurring upstream or downstream of P2X7 receptor activation. Activation of the P2X7 receptor by ATP leads to the formation of a pore, which permeabilizes the plasma membrane to molecules up to 900 Da including the dye YO‐PRO‐1 (Rassendren et al., 1997; Steinberg et al., 1987). YO‐PRO‐1 can be used as a readout of P2X7 receptor activation (Rat et al., 2017). WT pBMDMs were primed with LPS and stimulated with ATP in the presence of YO‐PRO‐1. Pre‐treatment with K2P inhibitor TPA, and general K^+^ channel inhibitor TEA, had no effect on P2X7‐dependent pore formation, but the P2X7 inhibitor, oxidized ATP (oATP) inhibited YO‐PRO‐1 uptake (Supplementary Figure S8). These data suggest that THIK‐1 regulated ATP‐induced NLRP3 activation downstream of P2X7 receptor activation.

Silica induced IL‐1β release was still inhibited by TPA in THIK‐1 KO BMDMs, indicating that TPA can also inhibit NLRP3 independently of THIK‐1 inhibition (Supplementary Figure S9), potentially by targeting TWIK‐2. We then used western blotting to further characterize the effect of THIK‐1 KO on NLRP3 activation. Caspase‐1, IL‐1β, and GSDMD processing were reduced in THIK‐1 KO BMDMs in comparison to WT in response to ATP (Figure 5b). Following our findings that TPA also inhibited NLRP3 priming we sought to clarify whether these effects of THIK‐1 KO were due to priming or activation. THIK‐1 KO had no effect of either IL‐6 or TNF release in response to LPS (Figure 5c). Using western blot, we confirmed knocking out THIK‐1 did not inhibit NLRP3 or pro‐IL‐1β protein expression stimulated by LPS (Figure 5d). These data suggest THIK‐1 is specifically required for ATP‐induced NLRP3 activation in pBMDMs but is dispensable for activation in response to other canonical stimuli and NLRP3 priming. Furthermore, these data suggest TPA inhibited NLRP3 activation and priming independently from inhibiting THIK‐1.

### 3.6 THIK‐1 regulates ATP and nigericin‐induced NLRP3 inflammasome activation in microglia

**FIGURE 6:**
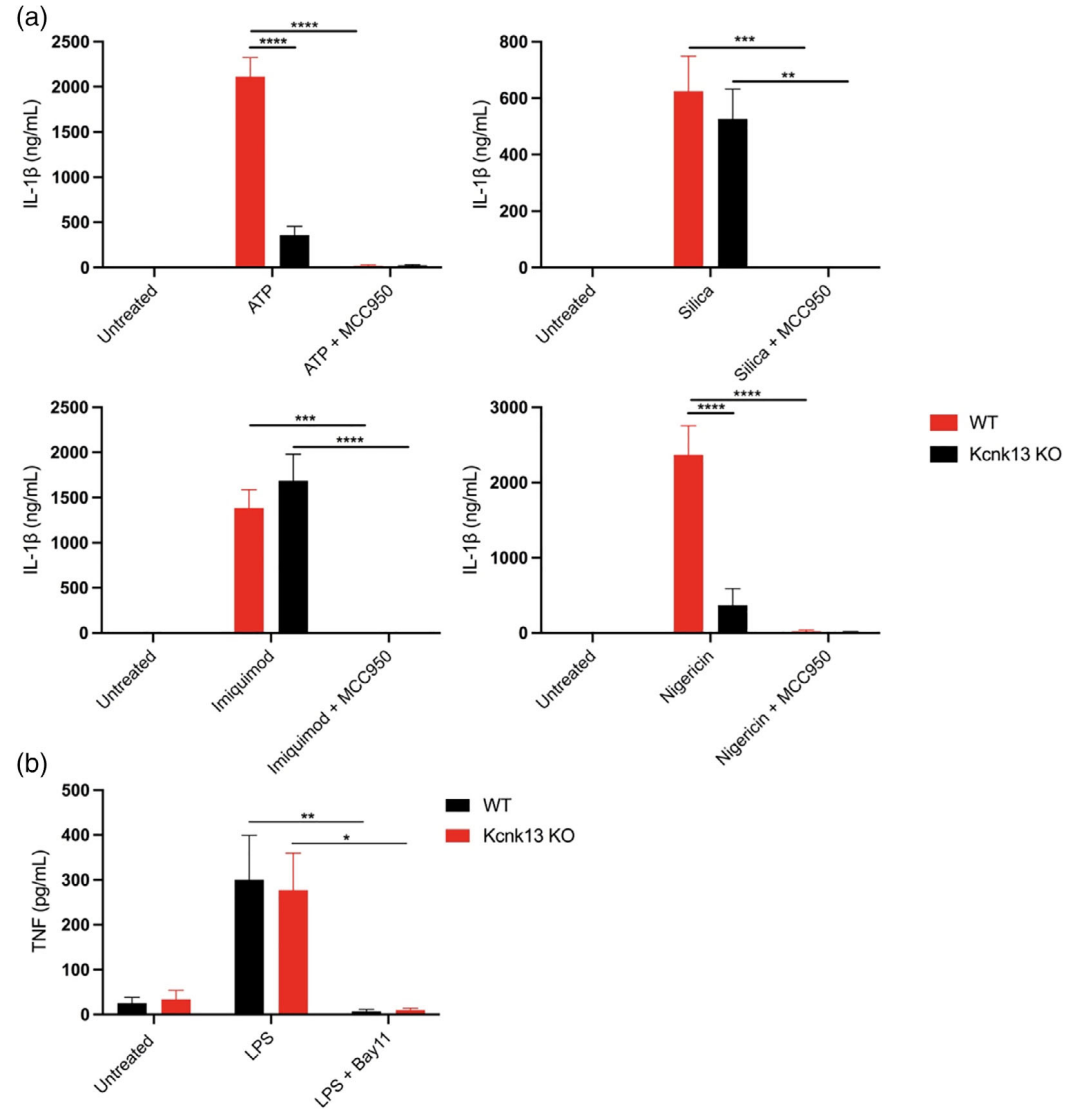
THIK‐1 regulates NLRP3 activation in primary adult microglia. (a) IL‐1β ELISA of the supernatant of primary wild‐type (WT) and Kcnk13 knockout (KO) adult microglia primed with LPS (1 μg ml^−1^, 4 h) followed by pretreatment with MCC950 (10 μM) for 15 min before stimulation with ATP (5 mM, 1 h) (*n* = 4), silica (300 μg ml^−1^,4 h) (*n* = 3), imiquimod (75 μM, 2 h) (*n* = 3) or nigericin (10 μM, 1 h) (*n* = 4). (b) TNF ELISA of the supernatant of primary wild‐type (WT) and Kcnk13 knockout (KO) primary adult microglia pretreated with Bay11(10 μM) for 15 min before priming with LPS (1 μg ml^−1^, 4 h) (*n* = 5). ****p < .0001, ***p < .001, **p < .01, *p < .05 determined by two‐way ANOVA with Bonferroni's post hoc analysis. Values shown are the mean ± SEM

We next harvested adult microglia from THIK‐1 KO mice to determine whether THIK‐1 regulates NLRP3 activation in microglia in addition to peripheral macrophages. WT and THIK‐1 KO microglia were primed with LPS and NLRP3 was activated by stimulation with ATP, silica, imiquimod and nigericin. Knocking out THIK‐1 inhibited NLRP3 activation in response to both ATP and nigericin but had no effect on silica or imiquimod‐induced activation (Figure 6a). One potential explanation for the different impact of THIK‐1 KO in microglia versus BMDMs following nigericin‐induced NLRP3 activation is the difference in THIK‐1 expression between the two cell types. THIK‐1 appears more highly expressed in microglia and may therefore play a broader role in NLRP3 activation when compared to BMDMs (Supplementary. Figure S1). Further, THIK‐1 KO had no effect on TNF release from microglia in response to LPS treatment (Figure 6b). These data suggest THIK‐1 regulates NLRP3 activation in brain resident microglia in addition to peripheral macrophages.

## 4 DISCUSSION

These data reveal THIK‐1 as a regulator of ATP‐induced NLRP3 activation and suggest that this may occur through a mechanism independent of ASC oligomerization. These data suggest that in response to extracellular ATP THIK‐1 is required for the activation of caspase‐1. Numerous studies have provided evidence that K^+^ efflux is a common event required for activation of NLRP3 in response to many stimuli (Muñoz‐Planillo et al., 2013; Pétrilli et al., 2007). Recent findings have identified K2P channels TWIK‐2 and THIK‐1 as potential regulators of the K^+^ sensitive process required for NLRP3 activation (Di et al., 2018; Madry et al., 2018). Knockdown of the TWIK‐2 channel prevents K^+^ efflux and NLRP3 activation induced by ATP while having no effect on K^+^ channel independent stimuli nigericin and imiquimod (Di et al., 2018). Furthermore, TWIK‐2 is required for sepsis‐induced NLRP3 inflammasome activation and inflammation in vivo (Di et al., 2018). Previous research has also associated an additional K2P channel, THIK‐1 with microglial function and ATP‐induced IL‐1β release in hippocampal slices (Madry et al., 2018). Genetic KO of THIK‐1 in mice results in depolarization of microglia, decreased microglial ramification, reduced microglial surveillance, and reduced IL‐1β release in response to ATP (Madry et al., 2018). Both studies suggest TWIK‐2 and THIK‐1 activation are regulated by purinergic receptors (P2X7 and P2Y12 respectively) (Di et al., 2018; Madry et al., 2018). In support of these previous findings, we show in this study pharmacological inhibition of K2P channels reduced NLRP3 activation in murine macrophages and microglia. Consistent with prior studies (Madry et al., 2018) we demonstrate in THIK‐1 KO macrophages that THIK‐1 is required specifically for ATP‐induced NLRP3 activation but is dispensable for activation in response to other canonical stimuli. These data, together with previous work, suggest THIK‐1 and TWIK‐2 regulate NLRP3 activation downstream of ATP‐induced activation of purinergic receptors.

It is well established that purinergic receptors play a role in NLRP3 activation. ATP activation of the P2X7 receptor induces K^+^ efflux and NLRP3 activation (Hafner‐Bratkovič & Pelegrín, 2018). Several studies suggest two complementing models for P2X7 cell permeabilization, the opening of a P2X7‐dependent pore, and P2X7 mediated opening of additional large conductance channels such as hemichannels and K^+^ channels (Browne et al., 2013; Di et al., 2018; Jiang et al., 2005; Pelegrín, 2011). Prior research has also suggested other purinergic receptors other than P2X7 can influence NLRP3 activation (Gombault et al., 2012). In particular, the P2Y family of G‐protein coupled receptors which are also stimulated by nucleotides such as ATP and ADP are associated with NLRP3 activation, suggesting P2Y receptors may also regulate NLRP3 activation (Baron et al., 2015; Riteau et al., 2012). Pharmacological inhibition of P2Y1 reduces nano‐particle‐induced NLRP3 activation (Baron et al., 2015). ADP and UTP may also induce NLRP3 activation via P2Y receptor activation (Riteau et al., 2012). It is therefore possible ATP, and its metabolites stimulate purinergic receptors which indirectly induce K^+^ efflux and NLRP3 activation through downstream opening of K^+^ channels. In this study, we observed K2P channel inhibition to reduce ATP‐induced NLRP3 activation without impacting P2X7 receptor activity suggesting THIK‐1 regulates NLRP3 activation downstream of P2X7 receptor activation. P2X7 depletion blocks ATP‐dependent NLRP3 activation and is thus fundamentally required for ATP‐induced NLRP3 activation (Solle et al., 2001). However, deletion of P2Y12 also reduces NLRP3 activation in response to ATP (Suzuki et al., 2020). Together, previous findings and this study suggest P2X7 and P2Y12 may both be required for ATP‐induced NLRP3 activation, potentially in part through regulation of K^+^ currents through K2P channels such as THIK‐1 and TWIK‐2. The findings that genetic ablation of THIK‐1 or TWIK‐2 inhibits NLRP3 activation in response to ATP suggests the two channels are non‐redundant in their regulation of the NLRP3 inflammasome. Therefore, indicating activation of both THIK‐1 and TWIK‐2 is required for the activation of the NLRP3 inflammasome in response to ATP signaling.

We recently reported a mechanism of NLRP3 activation in which a Cl^−^ ‐dependent step is required to drive NLRP3‐dependent ASC oligomerization (Green et al., 2018). Although Cl^−^ efflux was required to form an ASC speck, K^+^ efflux was required to permit activation of caspase‐1 (Green et al., 2018). These previous findings are supported by recent research which demonstrated low intracellular K^+^ levels trigger a conformational change in ASC oligomer structure resulting in enhanced caspase‐1 recruitment and activation (Martín‐Sánchez et al., 2020). In the present study, we show that inhibition of K2P channels, non‐selective K^+^ channel inhibition, and K^+^ efflux blockage, all inhibited caspase‐1 activation without blocking the formation of NLRP3‐dependent ASC specks in response to ATP. Furthermore, we show both inhibition of K^+^ and Cl^−^ efflux together abolished ATP‐induced speck formation. These data provide further evidence dissociating the impact of Cl^−^ and K^+^ efflux on NLRP3 formation and activation with Cl^−^ driving ASC oligomerization and K^+^ efflux dependent mechanism acting potentially via K2P channels driving caspase‐1 activation. These data suggest that K2P channels may be required to enable full activation of the inflammasome and caspase‐1 in response to ATP.

The present study identifies THIK‐1 as a regulator of NLRP3 activation in mouse macrophages and microglia in response to the canonical stimuli ATP. Consistent with previous work (Green et al., 2018) we also report that the formation of ASC specks can occur without downstream activation of caspase‐1 and IL‐1β cleavage. We show THIK‐1 is required for NLRP3 dependent caspase‐1 activation and IL‐1β release in response to ATP. These results demonstrate that multiple K^+^ channels may be involved in P2X7 dependent NLRP3 activation and highlight the therapeutic potential of targeting K^+^ channels to limit aberrant NLRP3‐induced inflammation in disease. THIK‐1 represents a viable therapeutic target for limiting NLRP3 inflammasome activation in peripheral and CNS diseases.

## AUTHOR CONTRIBUTIONS

Samuel Drinkall, Michael Harte, Catherine B Lawrence, and David Brough designed research; Samuel Drinkall, Samuel Russell, Clare Bender, performed research; David Brough, Michael Harte, Bernadino Ossola, Nicola B. Brice, Lee A. Dawson, contributed new reagents/analytic tools; Samuel Drinkall, Michael Harte, and David Brough analyzed data; and Samuel Drinkall, Michael Harte, Catherine B Lawrence, Bernadino Ossola, Lee A. Dawson, and David Brough wrote the article.

## Supporting information


**Appendix** S1: Supporting Information

## Data Availability

The data that support the findings of this study are available from the corresponding authors upon reasonable request.
