# Supplementary material for: The two pore potassium channel THIK‐1 regulates NLRP3 inflammasome activation
Source: Glia. 2022 Mar 30;70(7):1301–16. doi: 10.1002/glia.24174 (PMC9314991; doi:10.1002/glia.24174)
Supplement: Supplementary file 1 — Appendix S1: Supporting Information [file GLIA-70-1301-s001.docx]

**Supplementary Information for**

**The Two pore Potassium Channel THIK-1 Regulates NLRP3 Inflammasome Activation**

Samuel Drinkall^1^, Catherine B Lawrence^2^, Bernadino Ossola^5^, Samuel Russell^5^, Clare Bender^5^,

Nicola B. Brice^5^, Lee A. Dawson^5^, Michael Harte^1,4^*, David Brough^2,3,4^*

Michael Harte, David Brough

Email: michael.harte@manchester.ac.uk, david.brough@manchester.ac.uk

<


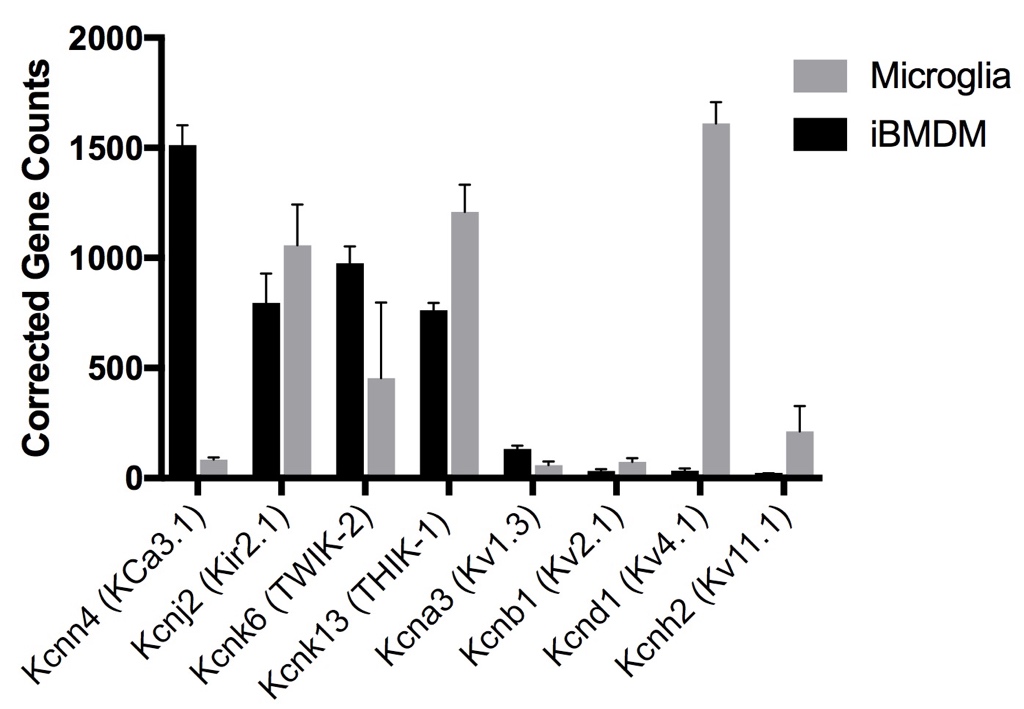


**Fig. S1. K+ Channels Expressed in Cultured Microglia and iBMDM Cells.** Data shows K+ channels expressed by untreated adult mouse microglia (n=6) and mouse iBMDMs following LPS treatment (1 μg mL^-1^, 2 h, n=4). Data were collected through data mining of RNA sequencing data bases collected from our research group on microglia (https://braininflammationgroup-universityofmanchester.shinyapps.io/NLRP3KOmicroglia) and iBMDMs (<https://braininflammationgroup-universityofmanchester.shinyapps.io/GrapheneOxide/>). Only genes with corrected counts above 80 are shown. Data are represented as mean corrected gene counts generated through normalizing raw gene counts relative to gene library sizes.


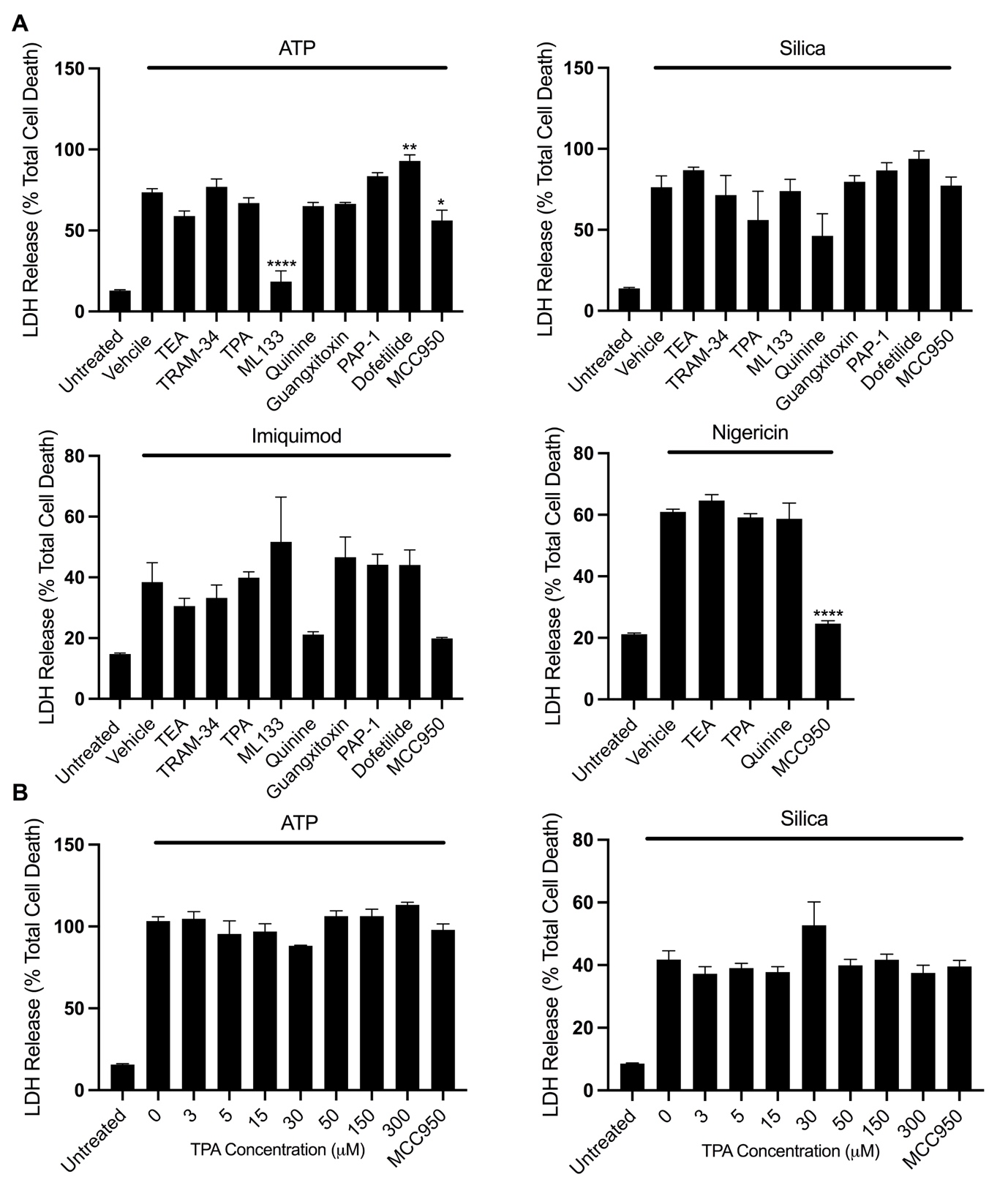


Fig. S2. K^+^ channel inhibitor impact on LDH release from pBMDM. (A) LDH release assay of the supernatant of pBMDMs primed with LPS (1 μg mL^-1^, 4 h) followed by pretreatment with MCC950 (10 μM) or K+ channel inhibitors TEA (50 mM), TRAM-34 (10 μM), TPA (50 μM), ML-133 (20 μM), Quinine (100 μM), Guangitoxin-1E (25 nM), PAP-1 (2 μM) or Dofetilide (1 μM) for 15 minutes before stimulation with ATP (5 mM, 1 h) (n=4), silica (300 µg mL^-1^, 4 h) (n=5), imiquimod (75 µM, 2 h) (n=3) or nigericin (10 µM, 1 h) (n=4). (B) LDH release assay of the supernatant of pBMDMs primed with LPS (1 μg mL^-1^, 4 h) followed by pretreatment with MCC950 (10 μM) or TPA (3-300 μM) before stimulation with ATP (5 mM, 1 h) (n=5) or Silica (300 µg mL^-1^, 4 h) (n=3). ****p<0.0001, **p<0.01, *p<0.05 determined by one-way ANOVA with Dunnett’s post hoc analysis. Values shown are the mean ± SEM.


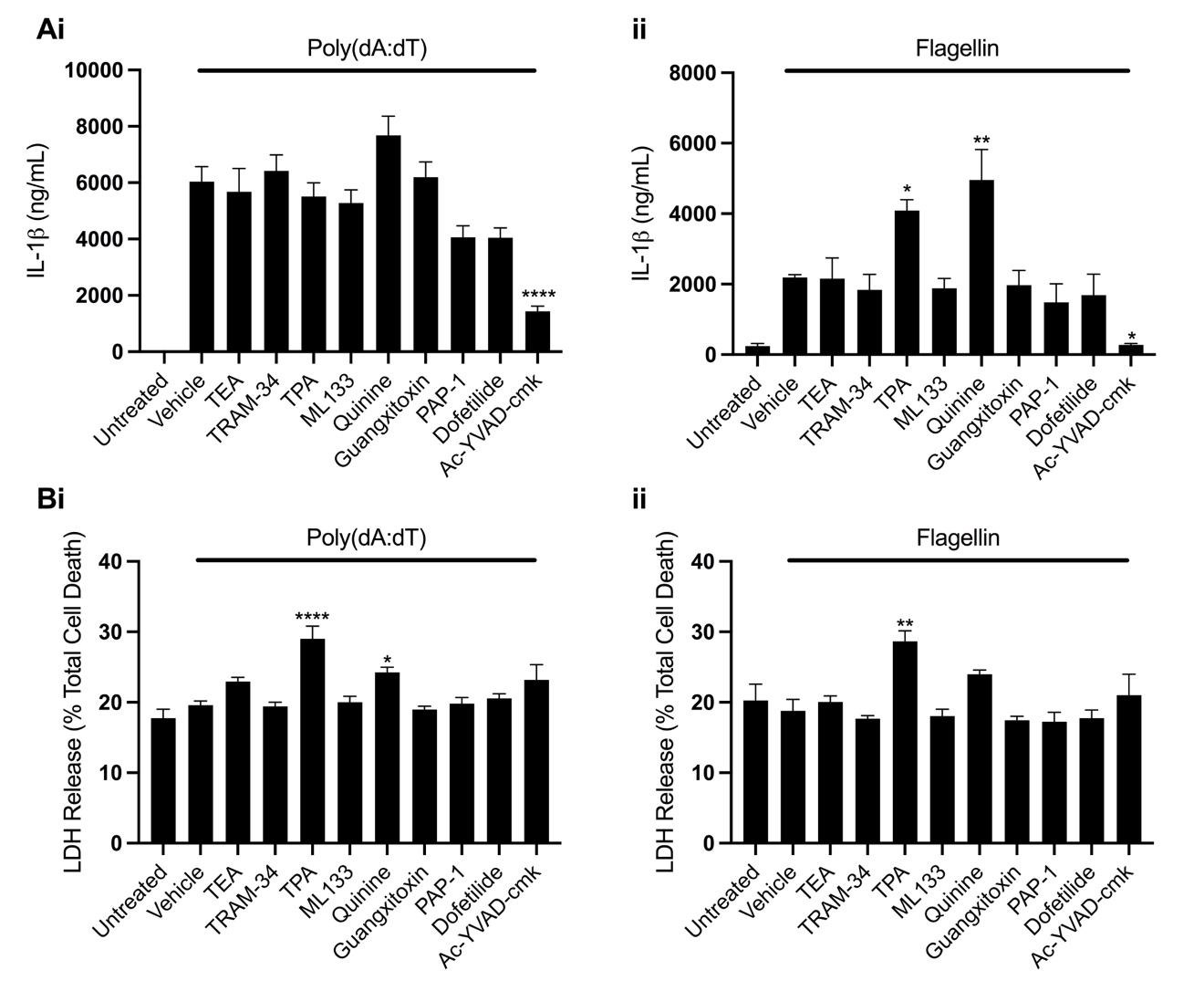


Fig. S3. Pharmacological inhibition of two pore domain potassium channels selectively regulates the NLRP3 inflammasome. (A) IL-1β ELISA and (B) LDH release assay of the supernatant of pBMDMs primed with LPS (1 μg mL^-1^, 4 h) followed by pretreatment with the caspase-1 inhibitor Ac-YVAD-cmk (50 μM) or K+ channel inhibitors TEA (50 mM), TRAM-34 (10 μM), Tpa (50 μM), ML-133 (20 μM), Quinine (100 μM), Guangitoxin-1E (25 nM), PAP-1 (2 μM) or Dofetilide (1μM) for 15 min before stimulation with transfected Poly(dA:dT) (1 μg mL^-1^, 4 h) (n=3) or transfected ultrapure flagellin from Salmonella typhurium (1 μg mL^-1^, 4 h) (n=3). *****p*<0.0001, ****p*<0.001, ***p*<0.01, *p<0.05 determined by one-way ANOVA with Dunnett’s post hoc analysis. Values shown are the mean ± SEM.


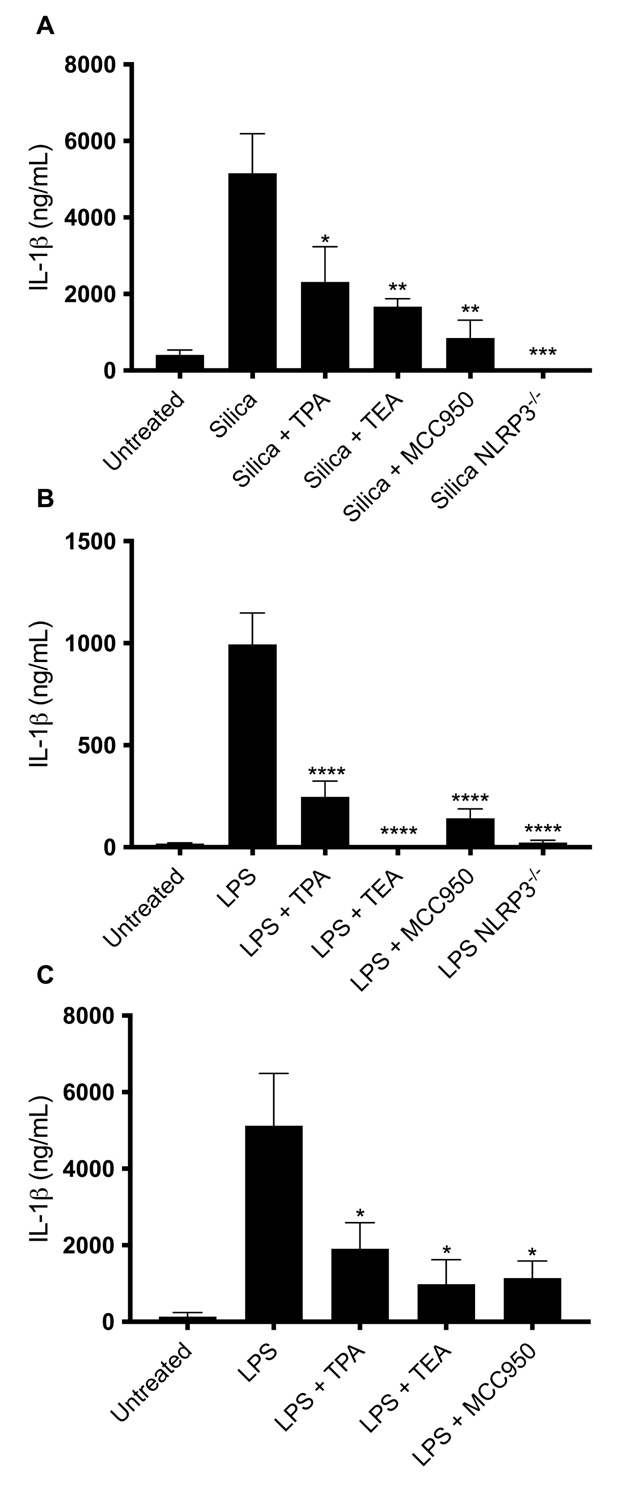


Fig. S4. Pharmacological inhibition of two-pore domain potassium channels blocks canonical and alternative NLRP3 activation in human monocytes. (A) IL-1β ELISA of the supernatant of WT and NLRP3 KO THP-1 monocytes primed with LPS (1 μg mL^-1^, 4 h) followed by pretreatment with vehicle control, Tpa (50 μM), TEA (50 mM) or MCC950 (10 μM) for 15 min before stimulation with silica (300 µg mL-1,4 h) (n=4). (B) IL-1β ELISA of the supernatant of WT and NLRP3 KO THP-1 monocytes pretreated with vehicle control, Tpa (50 μM), TEA (50 mM) or MCC950 (10 μM) for 15 min before stimulation with LPS (1 μg mL^-1^, 16 h) (n=6). (C) IL-1β ELISA of the supernatant of primary human monocytes pretreated with vehicle control, Tpa (50 μM), TEA (50 mM) or MCC950 (10 μM) for 15 min before stimulation with LPS (1 μg mL^-1^, 16 h) (n=3). *****p*<0.0001, ****p*<0.001, ***p*<0.01, **p*<0.05 determined by one-way ANOVA with Dunnett’s post hoc analysis. Values shown are the mean ± SEM.


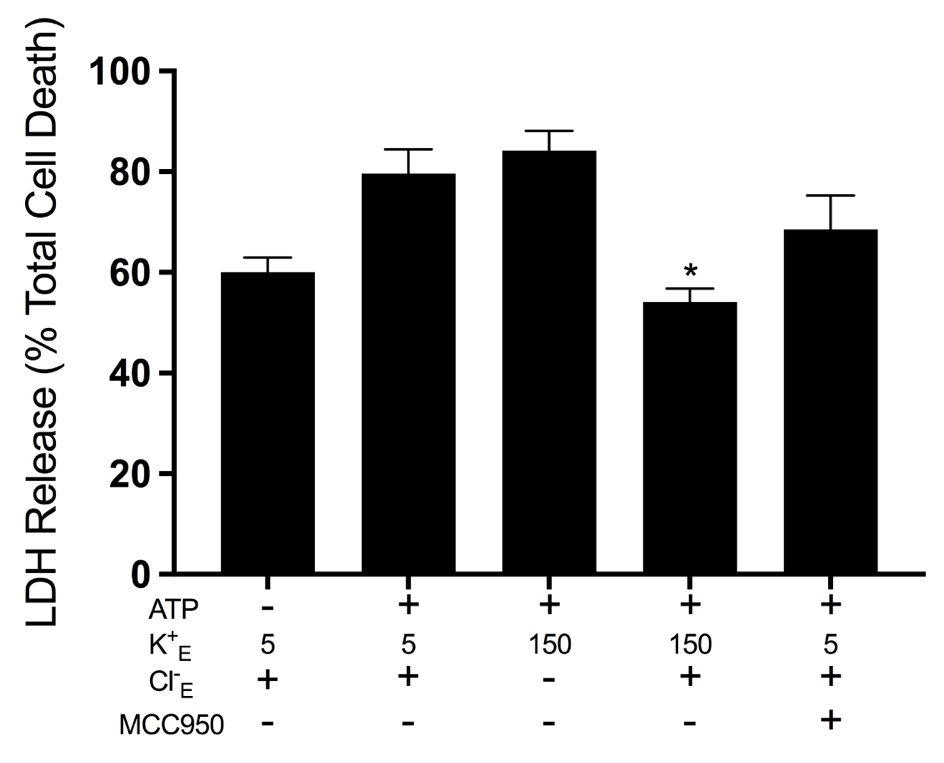


Fig. S5. Altered ionic K+ and Cl- solution impact on ATP induced cell death in pBMDM. LDH release assay of the supernatant of iBMDMs primed with LPS (1 μg mL^-1^, 4 h) followed by incubation in a control (145 mM NaCl/ 5 mM KCl), high K^+^ and normal Cl^-^ (150 mM KCl), high K^+^ and Cl^-^ free (150 mM KGluconate) or control and MCC950 (10 μM) solution for 15 minutes before stimulation with ATP (5 mM, 1 h) (n=6). *p<0.05 determined by one-way ANOVA with Dunnett’s post hoc analysis. Values shown are the mean ± SEM.


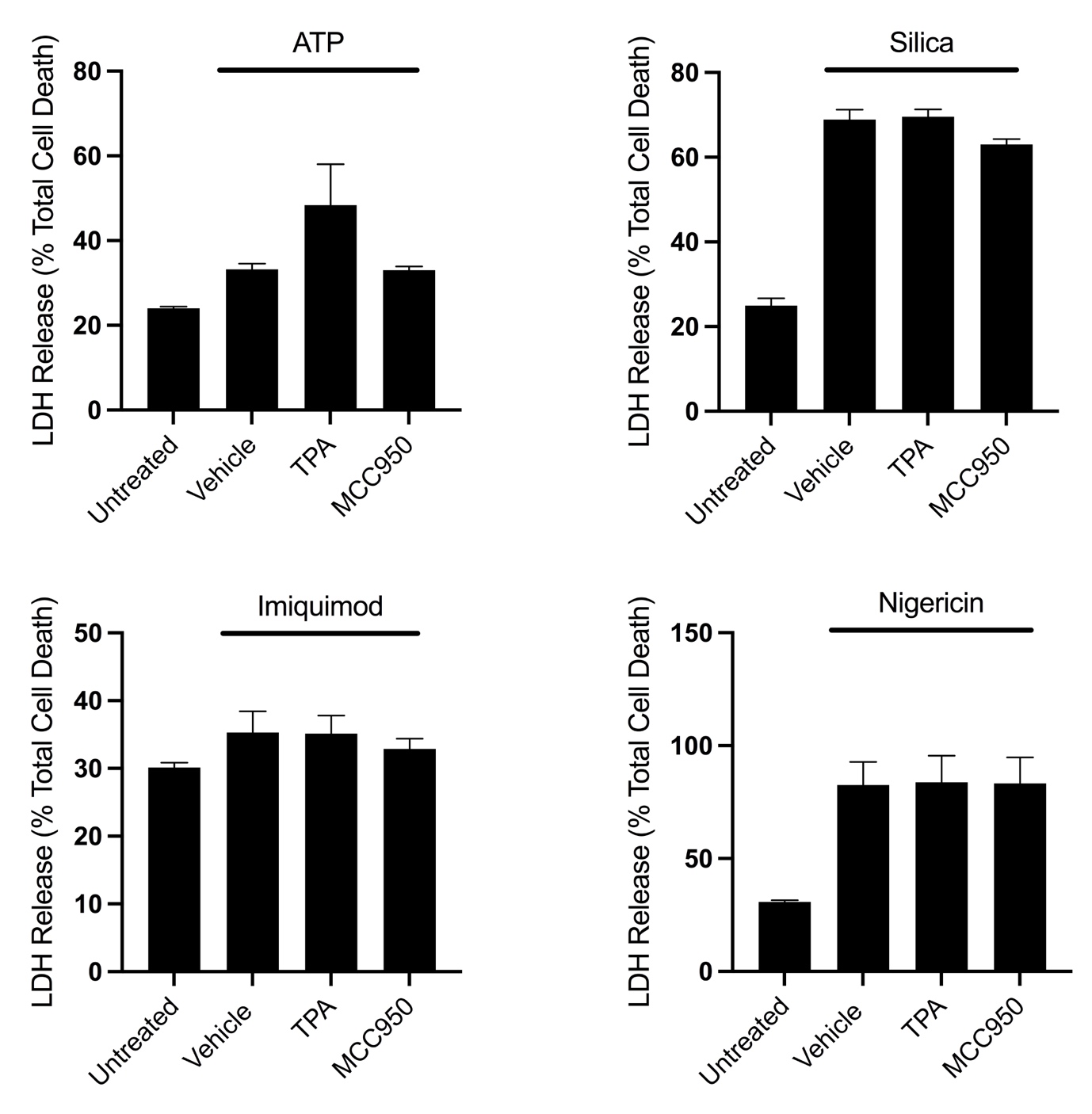


Fig. S6. Pharmacological inhibition of THIK-1 impact on LDH release from mixed glia. LDH release assay of the supernatant of primary mouse mixed glia primed with LPS (1 μg mL^-1^, 4 h) followed by pretreatment with vehicle control, TPA (50 μM) or MCC950 (10 μM) for 15 minutes before stimulation with ATP (5 mM, 1 h) (n=5), Silica (300 µg mL^-1^,4 h) (n=3), imiquimod (75 µM, 2 h) (n=4) or nigericin (10 µM, 1 h) (n=3). Significance determined by one-way ANOVA with Dunnett’s post hoc analysis. Values shown are the mean ± SEM.

**Fig. S7. THIK-1 knockout does not inhibit NLRP3 activation-induced LDH release.** LDH release assay of the supernatant of primary wild-type (WT) and Kcnk13 knockout (KO) BMDMs primed with LPS (1 μg mL^-1^, 4 h) followed by pretreatment with MCC950 (10 μM) for 15 minutes before stimulation with ATP (5 mM, 1 h) (n=4), silica (300 µg mL^-1^,4 h) (n=4), imiquimod (75 µM, 2 h) (n=4) or nigericin (10 µM, 1 h) (n=3), ***p<0.001, **p<0.01, *p<0.05 determined by two-way ANOVA with Bonferroni’s post hoc analysis. Values shown are the mean ± SEM.


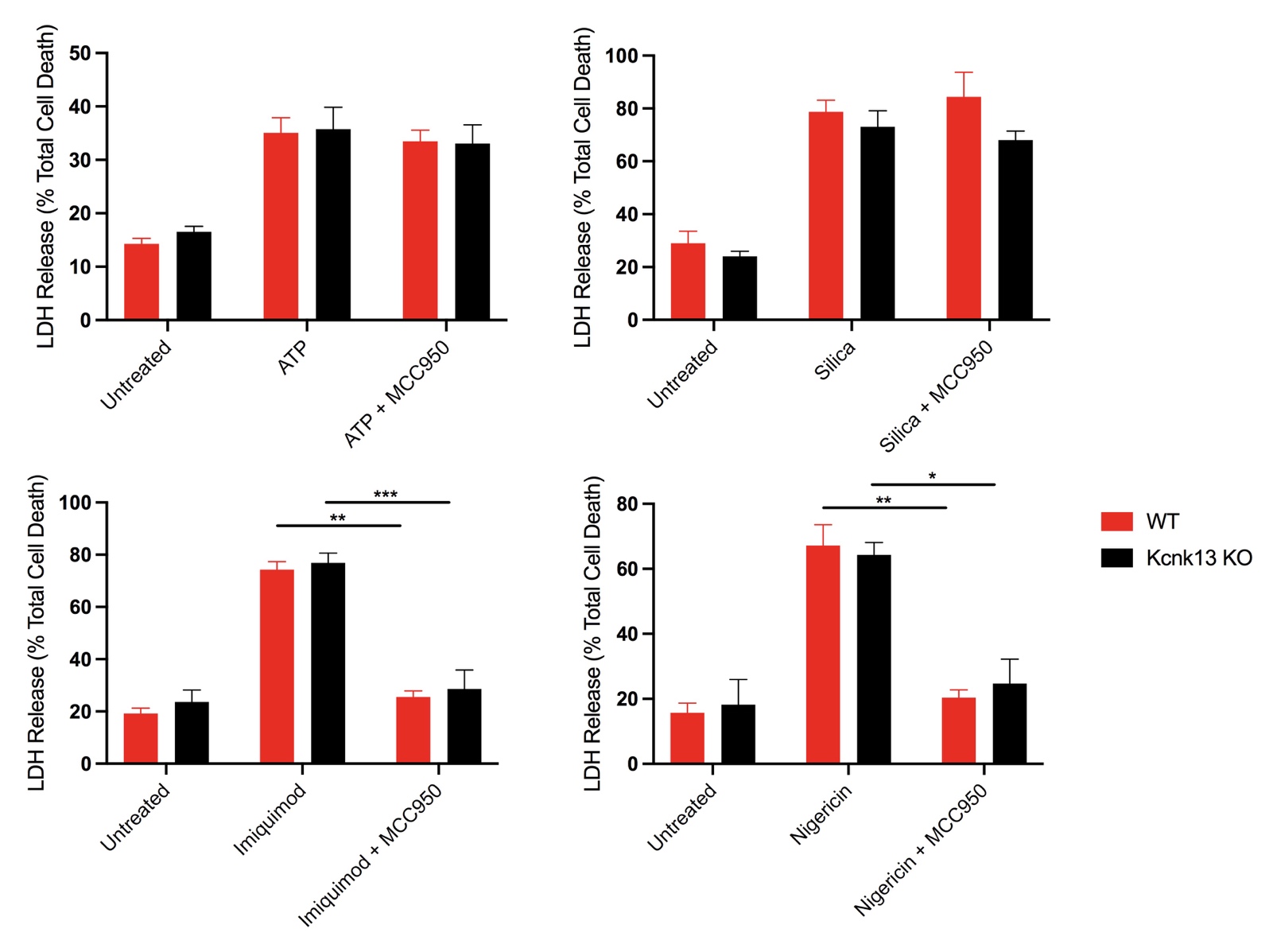

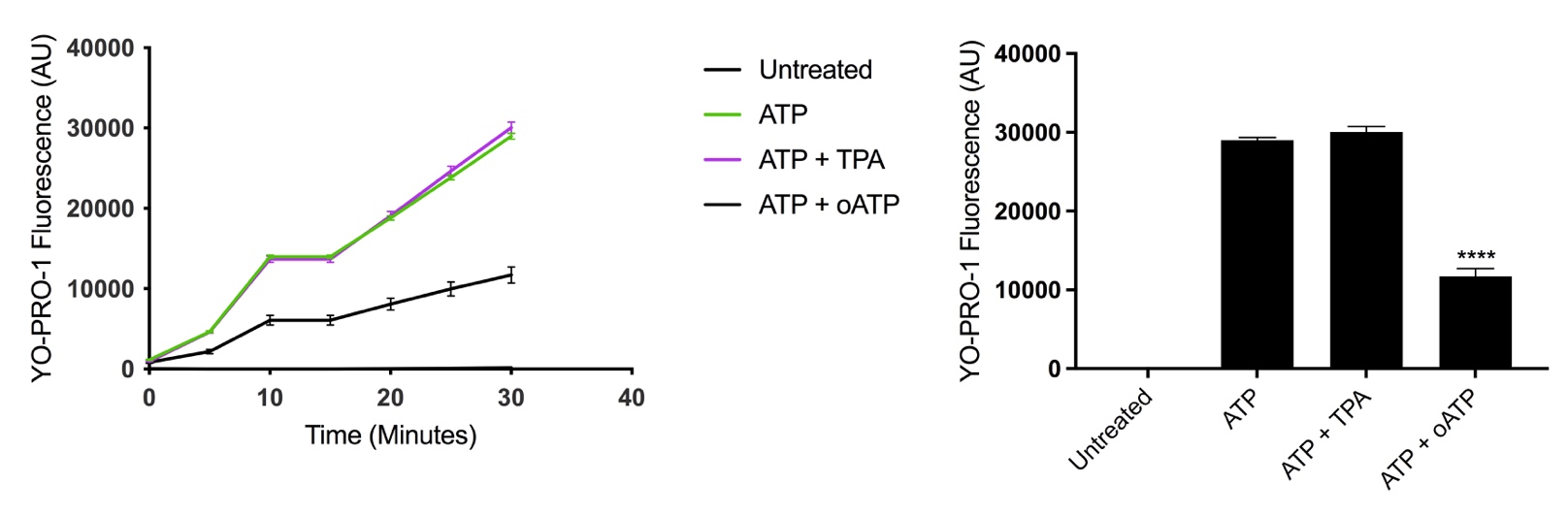


**Fig.S8. THIK-1 inhibition has no effect on ATP-induced P2X7 receptor activation. (**YO-PRO-1 fluorescent P2X7 assay over 30 minutes of pBMDMs primed with LPS (1 μg mL^-1^, 4 h) followed by pretreatment with TPA (50μM) or oATP (5 mM) for 15 minutes before stimulation with ATP (5 mM, 30 mins) (n=6), ****p<0.0001 determined by one-way ANOVA with Dunnett’s post hoc analysis. Values shown are the mean ± SEM.


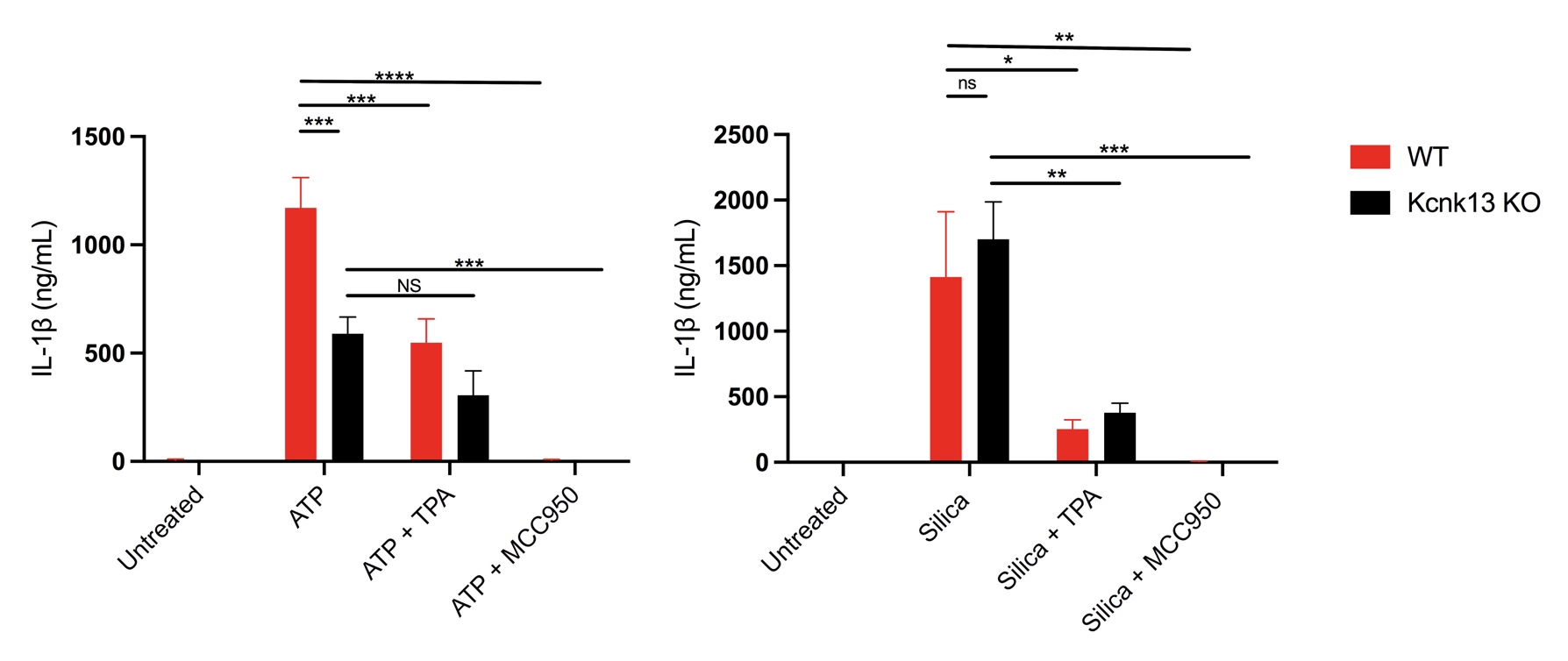


**Fig. S9. TPA inhibits the NLRP3 inflammasome independently from inhibiting the THIK-1 K^+^ channel.** IL-1β ELISA of the supernatant of primary wild-type (WT) and Kcnk13 knockout (KO) BMDMs primed with LPS (1 μg mL^-1^, 4 h) followed by pretreatment with vehicle control, TPA (50 μM) or MCC950 (10 μM) for 15 minutes before stimulation with ATP (5 mM, 1 h) (n=4), silica (300 µg mL^-1^,4 h) (n=4). ****p<0.0001, **p<0.01 **p<0.01, *p<0.05 determined by two-way ANOVA with Bonferroni’s post hoc analysis. Values shown are the mean ± SEM.
